# Molecular docking and dynamic simulation of marine natural products from soft coral-derived microbes against SARS-CoV-2 main protease and spike protein

**DOI:** 10.1038/s41598-026-37446-6

**Published:** 2026-02-11

**Authors:** Naga Venkata Anusha Anthikapalli, Vishal S. Patil, Phaniendra Alugoju, Vishwambhar V. Bhandare, Ankush Prasad, Sunil S. Jalalpure

**Affiliations:** 1 Department of Biophysics, Faculty of Science, Palacký University, Šlechtitelů 27, Olomouc, 77900 Czech Republic; 2 Department of Pharmacology, KLE College of Pharmacy, Belagavi, KLE Academy of Higher Education and Research, Deemed-to-be-University, Belagavi, Karnataka India; 3 Department of Microbiology, Shivaji University, Kolhapur, India

**Keywords:** Coral reefs, Severe acute respiratory syndrome coronavirus-2 (SARS-CoV-2), Coronavirus disease 2019 (COVID-19), Receptor binding domain (RBD), Spike protein, Molecular docking, Biochemistry, Computational biology and bioinformatics, Drug discovery, Microbiology

## Abstract

Coronavirus disease 2019 (COVID-19) is caused by the severe acute respiratory syndrome coronavirus-2 (SARS-CoV-2). Two key therapeutic target proteins of SARS-CoV-2, the Spike (S) protein and the main protease (Mpro), facilitate the entry of virus and its replication inside the host cell, respectively. Notably, several pointmutations in the receptor-binding domain (RBD) of the S-protein have led to the origin of different SARS-CoV-2 Variants of Concern (VOCs) including Alpha (B.1.1.7), Beta (B.1.351), Delta (B.1.617.2), Gamma (P.1), and Omicron (B.1.1.529). The low efficacy of currently available antiviral drugs against these VOCs highlights the need for screening and discovery of novel natural compounds against COVID-19. This study performed molecular docking of marine natural products from soft coral-derived microbes against the Mpro and the RBD of the S-protein from wild type (WT) and five VOCs. Many of the test compounds [e.g., Cottoquinazoline B and D (CQB/D), Tetraorcinol A (TOA), Versicoloritide A and C (VCA/C), Fumiquinazoline K, and Pencillanthranin A) showed stronger binding affinities compared to control antiviral drugs (nelfinavir and remdesivir) and formed favorable interactions with both Mpro and the RBD of S-protein. ADMET analysis revealed that most of the best-docked compounds obey Lipinski rule of five. Molecular dynamics (MD) simulation (200 ns) analysis further revealed stable binding conformations of the top docked complexes of (1) CQB with Mpro, (2) CQB with the RBD of WT S-protein, (3) TOA with the RBD of S-protein from beta variant (4) TOA with the RBD of S-protein from Omicron variant, (5) TOA with the RBD of S-protein from Delta variant, (6) TOA with the RBD of S-protein from Gamma variant, and (7) VCA with the RBD of alpha variant. However, future in vitro and in vivo studies are still required to validate efficacy of these compounds.

**Supplementary Information:**

The online version contains supplementary material available at 10.1038/s41598-026-37446-6.

## Introduction

The Severe Acute Respiratory Syndrome Coronavirus 2 (SARS-CoV-2) is an extremely infectious virus first discovered in 2019 in Wuhan, China^1^. It is likely to have originated from bat-derived coronavirus and transmitted to humans. The phylogenetic analysis of SARS-CoV-2 revealed that it shares more than 90% similarity to a bat SARS-like coronavirus. It causes a deadly respiratory syndrome called Coronavirus disease 2019 (COVID-19). The World Health Organization (WHO) declared COVID-19 as a pandemic in March 2020. More than 6.9 million deaths have been confirmed globally due to COVID-19. COVID-19 is usually characterized by mild to severe infections causing diverse ill effects such as sore throat, high fever, cough, headache, fatigue, hypoxemia, diarrhea, dyspnea, lymphopenia, acute cardiac injury, rhinorrhea, sneezing, pneumonia, and even death^2^. SARS-CoV-2 is an RNA virus and its total genome size ranges from 29.8 kb to 29.9 kb. The viral RNA genome encodes four vital structural proteins such as Spike (S), Envelop (E), Nucleocapsid (N), and Membrane Protein (M), and sixteen non-structural proteins (Nsp1–Nsp16)^3^. The Spike protein (S) and the Main protease (Mpro) encoded by Nsp5, are considered as important virulent factors because they promote host cell interaction and replication of the virus^4^.

The name corona is given due to the presence of a highly conserved Spike protein (S), a glycoprotein located on the envelope of the SARS-CoV-2. The Spike protein is a trimeric structure (made of three identical parts) that protrudes from the virus surface and is essentially involved in the entry of the virus particle into the host^5^. It binds to the host cell surface receptor, the human angiotensin-converting enzyme 2 (hACE2)^3,6–10^. Following the attachment of the virus to ACE2, the host cell proteases cleave the S protein into S1 and S2 subunits. The S1 subunit binds to the hACE2 receptor and forms a strong complex, thus S1 subunit acts as the main receptor binding domain (RBD). On the other hand, the S2 subunit helps in the fusion of the virus to the host cell membrane^11^. The six key amino acid residues of the RBD including L455, F486, Q493, S494, N501, and Y505, play a critical role in the attachment of the S-protein to hACE2^12^. Therefore, preventing the entry of virus by targeting the S-protein is considered an effective strategy to prevent SARS-CoV-2 infections^13^.

Anti-viral drugs (e.g., remdesivir, favipiravir, lopinavir, ritonavir, and ribavirin) to have potency against COVID-19 were found to be less effective in patients with comorbidities^14^. Hence, there is a growing interest in finding novel therapeutic antiviral compounds. Marinenatural compounds are potent sources of therapeutic drugs among other natural products^15^. Natural products derived from marine sponges, fungi, bacteria, plants, and soft corals have been reported to possess multiple pharmacological activities including antimicrobial activity^16,17^. Coral reefs are one of the main types of reefs in the ocean which are constructed by stony corals, through gradual accumulation of calcium carbonate skeleton. About 1/6th of the coastal line is bordered by coral reefs. Indeed, coral reefs support the life of about 1/4th of the marine organisms and additionally they help reducing the impact of floods and storm^18^.

Coral-microbes are considered the principal producers of marine natural products (MNPs). Coral reef microbes have gained considerable attention recently due to their ability to synthesize diverse secondary metabolites. However, there are limitations, such as the finite availability of coral resources and the challenges associated with culturing these microorganisms. Coralassociated fungi produce a wide range of secondary metabolites such as alkaloids, terpenes, cyclic peptides, lactones, sterols, mycotoxins, polyketides, and pyrones. The presence of different classes of metabolites highlights the importance of coral-associated microbes as a valuable resource for drug discovery. Currently, some of the drugs derived from marine sources are in clinical use. Most approved marine compounds are anti-neoplastic.. Marine drugs have diverse structural characteristics and mechanisms of action. A large number marine compounds are being tested in several pre-clinical and clinical studies^19^.

Several variations of SARS-CoV-2 have evolved over time due to five point mutations (N501Y, E484K/Q, K417N/T, L452R and T478K) in the RBD of the spike protein. The most widely reported SARS CoV-2 variants of concern (VOCs) include Alpha (B.1.1.7), Beta (B.1.351), Delta (B.1.617.2), Gamma (P.1), and Omicron (B.1.1.529)^20^. The SARS CoV-2 infections are still haunting and the global concern, which makes it challenging to develop effective vaccines and antiviral drugs against this life-threatening viral infections^21^. In silico computational approaches such as molecular docking have always been valuable for screening of large number of small molecules in a very short time, expediting drug discovery process^22,23^. SARS CoV-2 Mpro is essential for viral replication. Although Mpro has few mutations, they are not actually associated with the virulence of the virus or the large-scale infections. Whereas, S-protein shows different point-mutations and the resulting mutants are strongly associated with the severe viral infections, transmissibility, immune escape, and virulence capacity. In this context, this study focused on screening against Mpro (but not its mutants) and S-protein. We performed molecular docking of 119 MNPs from soft coral-derived microbes against SARS-CoV-2 main protease (Mpro), the RBD of wild type spike protein and five different spike variants of concern (VOCs) using Autodock vina software. Furthermore, molecular dynamics (MD) simulations were carried out for 200 ns to validate the binding stability of the best-docked complexes of coral-associated microbial natural compounds with the Mpro and RBD of WT and VOCs.

## Methods

### Selection and Preparation of ligands

**Fig. 1:**
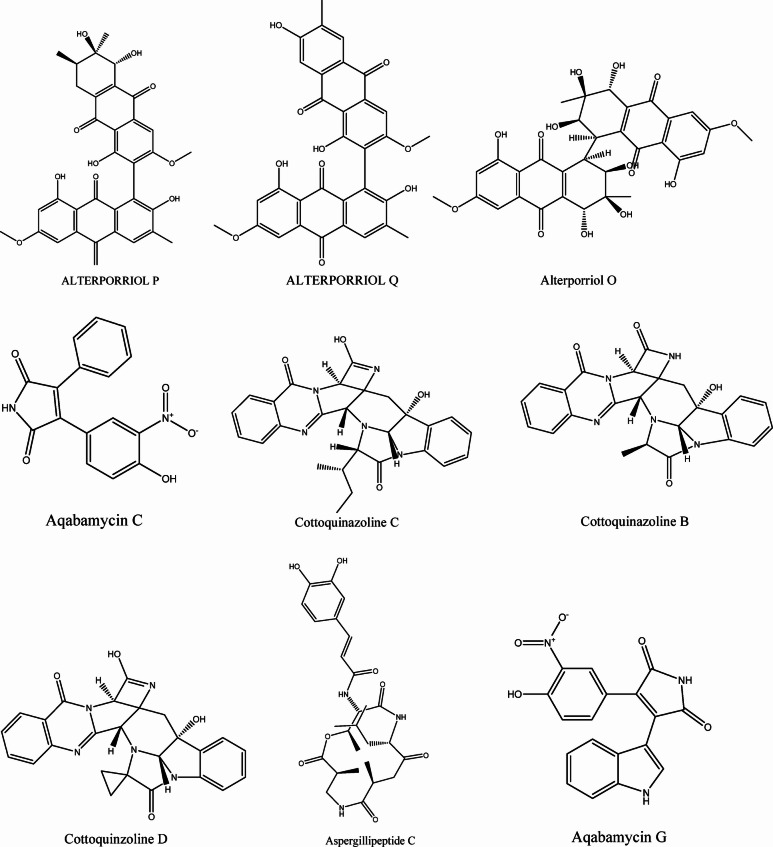

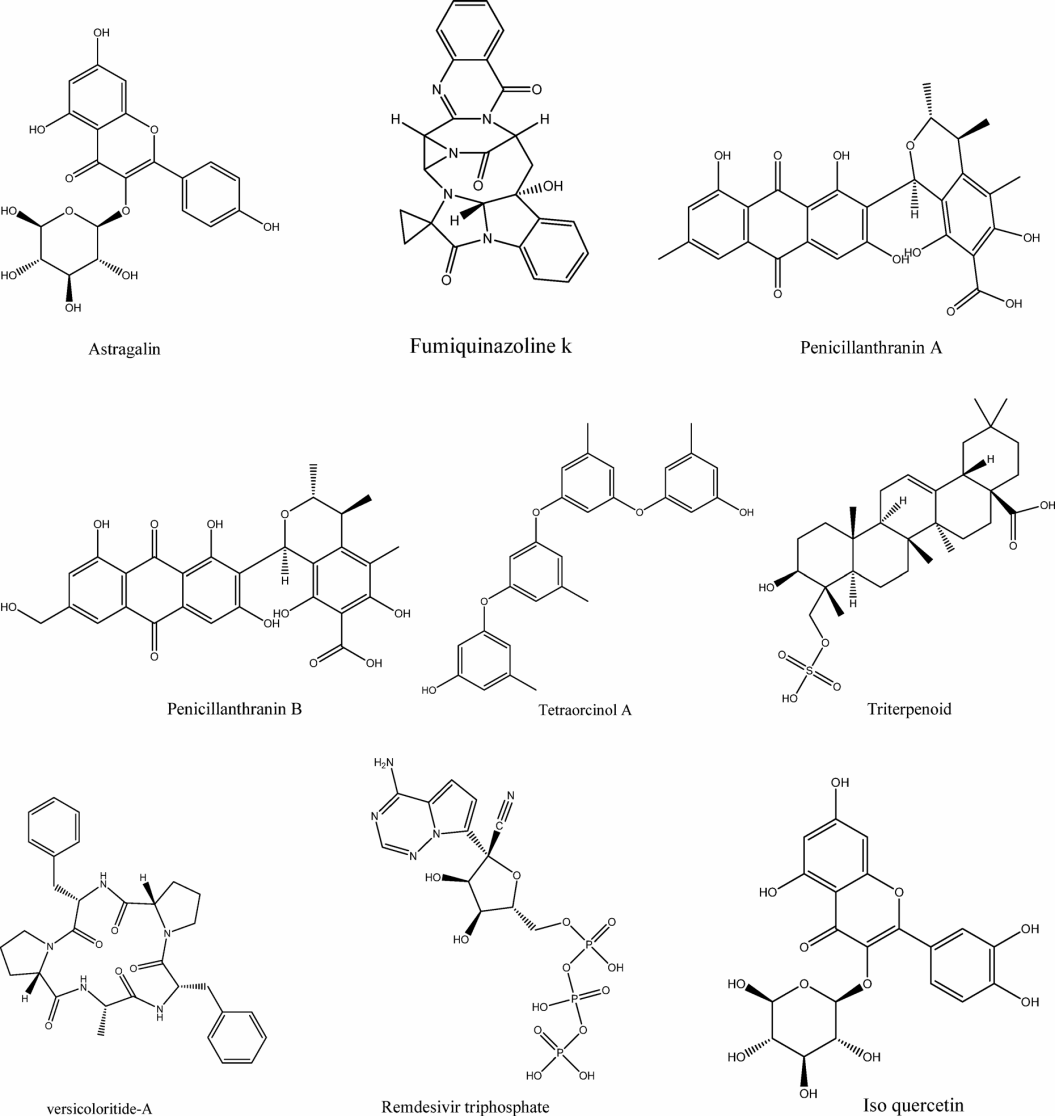
2D structures of MNPs from soft coral-derived microbes (Only few are here) including control ligand Remdesivir.

A total of 119 marine natural products from soft coral-derived microbes were selected from previous literature^24^ and their structures were retrieved from the PubChem database^25^. The three-dimensional structures of the screened compounds were prepared by addition of polar hydrogens, assigning Gasteiger charges, and performing energy minimization in UCSF Chimera 1.16 with default parameters^26^. The 2D structures of few MNPs screened were shown in Fig. 1.

### Prediction of physicochemical properties

The physiochemical properties of chemical compounds such as the number of H-bond donors (No. of HBD) and acceptors (No. of HBA) present, polar surface area (Mol PSA), lipophilicity (Mol LogP), and solubility (Mol LogS)of test compounds were predicted using Molsoft tools (https://www.molsoft.com/mprop/). Additionally, ADMETlab 2.0 was used to predict ADMET properties such as absorption, distribution, metabolism, excretion, and toxicity (https://admetmesh.scbdd.com/)^27^.

### Selection and Preparation of target proteins

The three-dimensional crystal structures of SARS-CoV-2 main protease (Mpro) and spike (S) protein of WT and other variants of concern (VOCs) were retrieved from the Protein Data Bank (PDB) data base (https://www.rcsb.org/)^25^. A list of PDB IDs and X-ray resolutions of target proteins used in this study are given in the Table 1. Using Chimera, missing parameters such as atoms, residues, missing loops, and side chains were checked and inserted. All water molecules (except the one near the substrate binding site) and non-protein residues were removed via structure optimization and energy minimization using Chimera^1,26^.

**Table 1:** List of selected target proteins used in this study.

PDB ID	Target Protein Name	X-ray resolution
6LU7	The crystal structure of COVID-19 main protease in complex with an inhibitor N3	2.16 Å
6M0J	Crystal structure of SARS-CoV-2 spike receptor-binding domain bound with ACE2	2.45 Å
7LWS	UK (B.1.1.7) SARS-CoV-2 S-GSAS-D614G variant spike protein in the 3-RBD-down conformation	3.22 Å
7LYK	South African (B.1.351) SARS-CoV-2 spike protein variant (S-GSAS-B.1.351) in the 2-RBD-up conformation	3.65 Å
7T9J	Cryo-EM structure of the SARS-CoV-2 Omicron spike protein	2.79 Å
7V8B	SARS-CoV-2 S-Delta variant (B.1.617.2) RBD and Angiotensin-converting enzyme 2 (ACE2) ectodomain	3.20 Å
7V82	Cryo-EM structure of SARS-CoV-2 S-Gamma variant (P.1) in complex with Angiotensin-converting enzyme 2 (ACE2) ectodomain, three ACE2-bound form conformation 1	2.80 Å

### Protein-ligand docking

Docking studies were carried out using Autodock Vina as explained in our previous study^28^. The ligand-binding site was positioned at the center of the grid box with the dimensions of the XYZ axes fixed at 30Å×30Å×30Å and a default exhaustiveness of 8. The size of the grid box and the XYZ dimensions were included in a configuration file (config file). Docking was performed by using Autodock Vina version 1.1.2 command line interface. Only nine binding modes are reportedin the results. Following docking, a log file consisting of binding modes and corresponding binding energies of coral reef compounds was generated. The binding modes were analyzed using BIOVIA Discovery studio visualizer 2021 and all non-bonded interactions were tabulated^29^.

### Molecular dynamics

The top-ranked docking conformations for target proteins, Mpro and spike protein were selected for all-atom MD simulations, conducted for 200 nanoseconds in an explicit solvent environment to evaluate their structural stability and intermolecular interactions. In the case of mutants, only the truncated portion (between 334–520 amino acids) of the S-protein from each of the VOCs was employed for MD simulations. This truncated portion corresponds to the RBD and does not consist of any missing residues. Such a targeted approach not only substantially reduces the computational costs but also facilitates a focused understanding of dynamic interactions between the RDB of spike VOCs and the test compounds. As described previously, MD simulations were carried out using GROMACS 2021.3 suite (https://www.gromacs.org/), employing the Amber ff99SB-ildn force field^30^. Partial atomic charges for the small-molecule ligands were assigned using the Antechamber tool with the ‘bcc’ charge model, while ligand topology and system parameter files were generated via the XLeap module of AmberTools (https://ambermd.org/AmberTools.php). Protein-ligand complex was solvated in a TIP3P water model within a rectangular box, ensuring a 10.0 Å buffer zone surrounding the solute. Counter-ions were added to neutralize the net charge of the system. Initial energy minimization was conducted using the steepest descent method followed by conjugate gradient minimization to achieve low-energy conformations. System equilibration was performed in two phases: under the NVT (constant number of particles, volume, and temperature) ensemble using the modified Berendsen thermostat to maintain a temperature at 300 K, and under the NPT (constant number of particles, pressure, and temperature) ensemble using the Parrinello-Rahman barostat to stabilize pressure at 1 bar. Long-range electrostatic and van der Waals interactions were treated using the Particle Mesh Ewald (PME) method with a 1 nm cut-off. Bond constraints were enforced using the LINCS (LINear Constraint Solver) algorithm. Post-simulation trajectory analyses were conducted using GROMACS built-in tools.

## Results

### Molecular Docking analysis of MNPs from soft coral-derived microbes against Mpro and the RBD of the wild-type spike protein

**Fig. 2:**
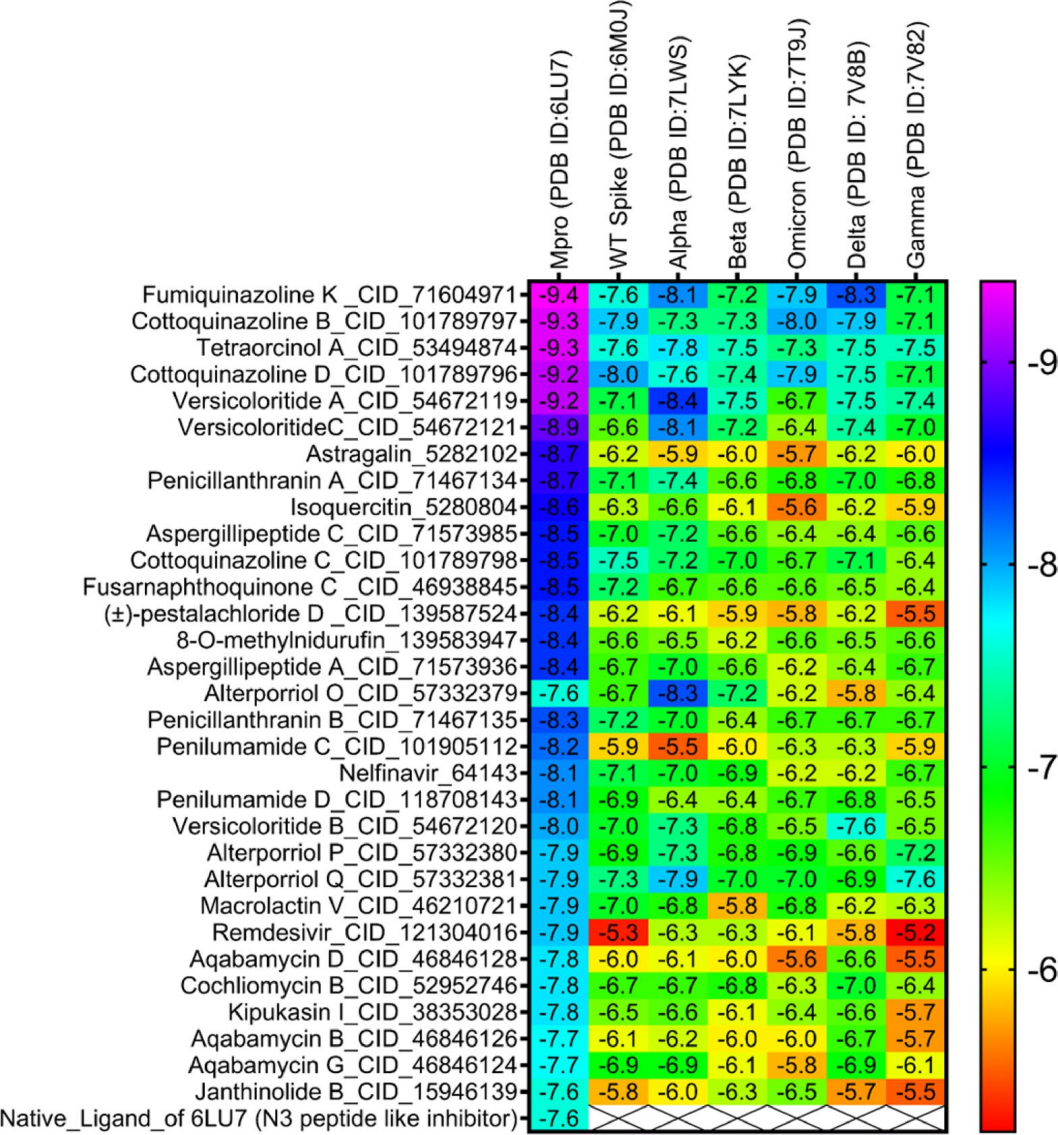
The heatmap represents the binding energies (kcal/mol) of the some of the top docked coral -associated microbial compounds docked with the SARS-CoV-2 main protease (Mpro, PDB ID: 6LU7), WT spike protein (6M0J), and spike protein variants from major VOCs (Alpha-PDB ID: 7LWS; Beta-PDB ID: 7LYK 7T9J_Omicron; 7V8B_Delta; 7v82_Gamma).

In this study, we chose 119 MNPs from coral-associated microbes for molecular docking against Mpro (PDB ID: 6LU7) and the RBD of S-protein (PDB ID: 6M0J). As positive control ligands, two anti-viral drugs Nelfinavir (NEF) and Remdesivir (REM) and two natural antiviral compounds astragalin and Isoquercetin, were chosen for docking against selected target proteins. All positive control ligands showed binding free energies in the range of −2.6 to −8.0 Kcal/mol and − 2.8 to −9.4 towards 6M0J and 6LU7, respectively. The binding energies of Fumiquinazoline K towards 6M0J and 6LU7 were found to be −7.6 and − 9.4 Kcal/mol, respectively. The binding energies of Cottoquinazoline B towards 6M0J and 6LU7 was found to be −7.9 and − 9.3 Kcal/mol respectively. The binding energy of Tetraorcinol A towards 6M0J and 6LU7 was found to be −7.6 and − 9.3 Kcal/mol respectively. The binding energy of Cottoquinazoline D towards 6M0J and 6LU7  was found to be −8.0 and − 9.2 Kcal/mol, respectively. The binding energy of Versicoloritide A towards 6M0J and 6LU7 was found to be −7.1 and − 9.2 Kcal/mol, respectively. Screened marine natural compounds with binding energies higher than the control ligands and the native ligand (N3) of Mpro, were only included in the heatmap (Fig. 2), with the lowest binding energy values in purple and the highest binding energy values in red. The physicochemical properties of the best-docked MNPs are given in Table 2.

Compounds such as Fumiquinazoline K (−9.4 Kcal/mol), Cottoquinazoline B (−9.3 Kcal/mol), Tetraorcinol A (−9.3 Kcal/mol), Cottoquinazoline D (−9.2 Kcal/mol), Versicoloritide A (−9.2 Kcal/mol), Versicoloritide C (−8.9 Kcal/mol), Pencillanthranin A (−8.7 Kcal/mol), and Astragalin (−8.7 Kcal/mol) showed strong affinities towards Mpro (6LU7) with binding energies in the range of −8.1 to −9.4 Kcal/mol compared to native ligand (N3) and the control antiviral drugs. While compounds such as Cottoquinazoline D (−8.0 Kcal/mol), Cottoquinazoline B (−7.9), Fumiquinazoline K (−7.6 Kcal/mol), Tetraorcinol A (−7.6 Kcal/mol), Cottoquinazoline C (−7.5 Kcal/mol), Alterporriol Q (−7.3 Kcal/mol), Fusarnaphthoquinone C (−7.2 Kcal/mol), Pencillanthranin B (−7.2 Kcal/mol), Versicoloritide A (−7.1 Kcal/mol), Pencillanthranin A (−7.1 Kcal/mol), Nelfinavir (−7.1 Kcal/mol) showed strong affinities towards WT S-protein (6M0J) with binding energies in the range of −7.1 to −8.0 Kcal/mol compared to the native ligand (N3) and control drugs.

**Fig. 3:**
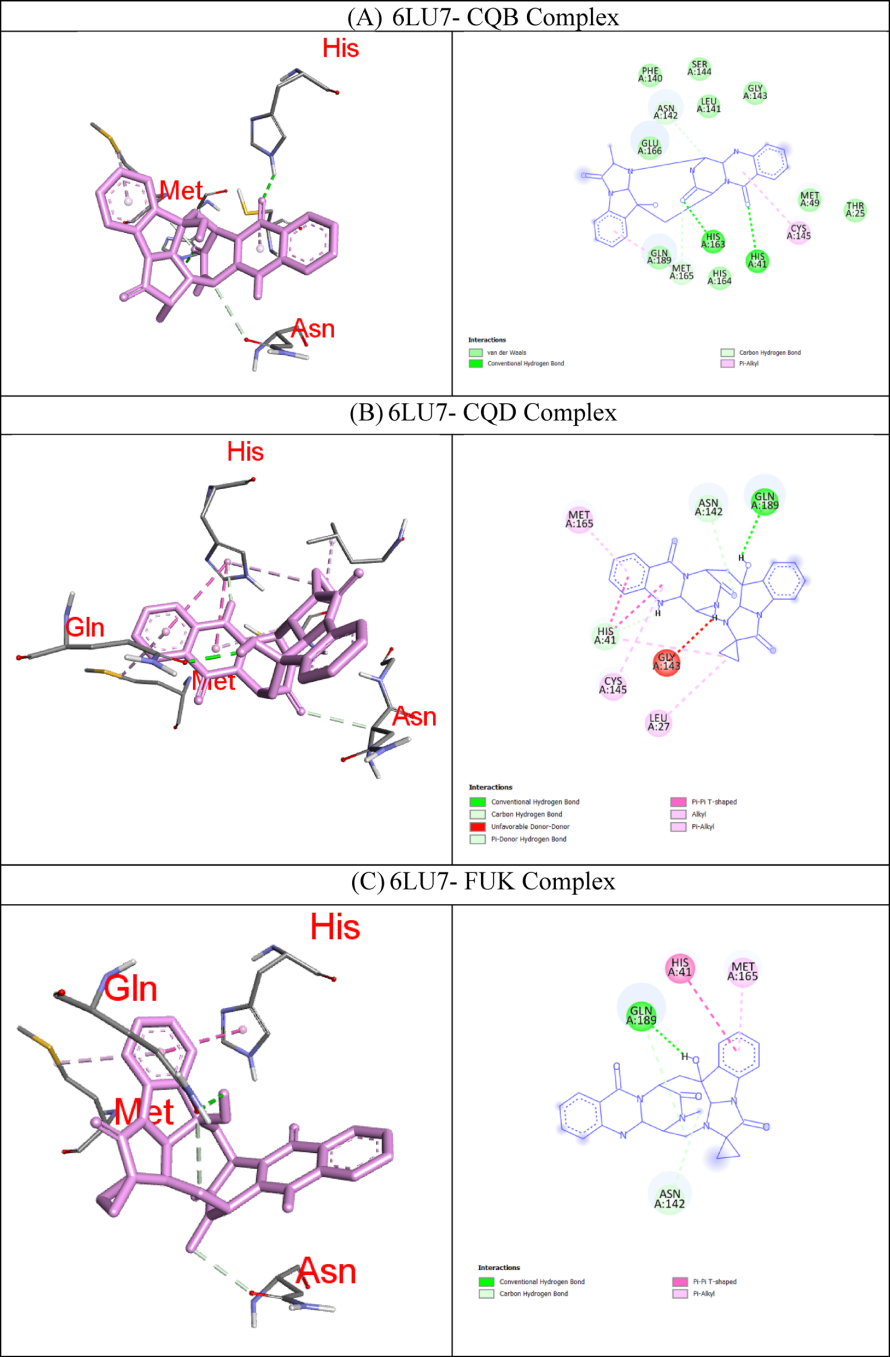

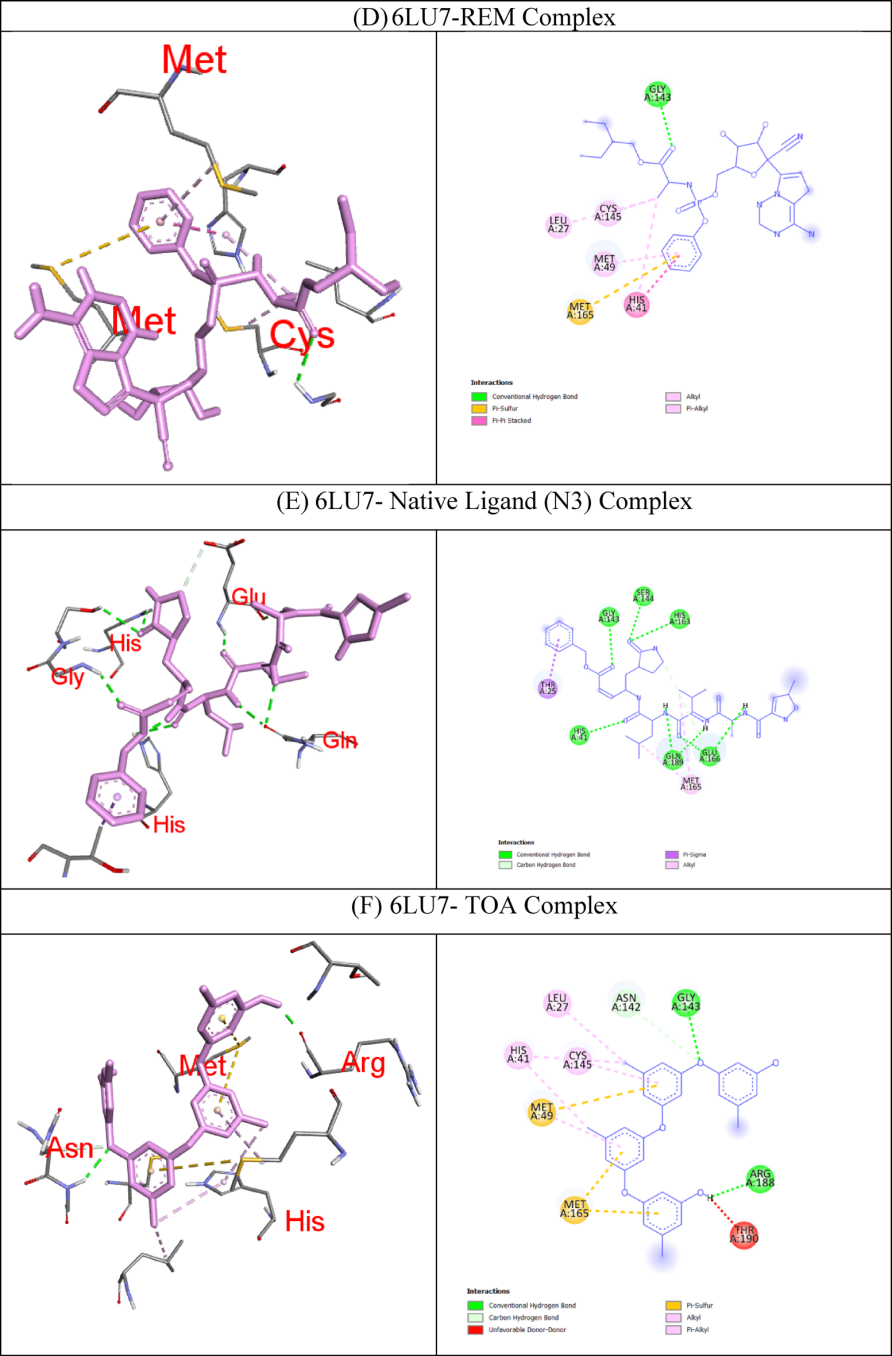

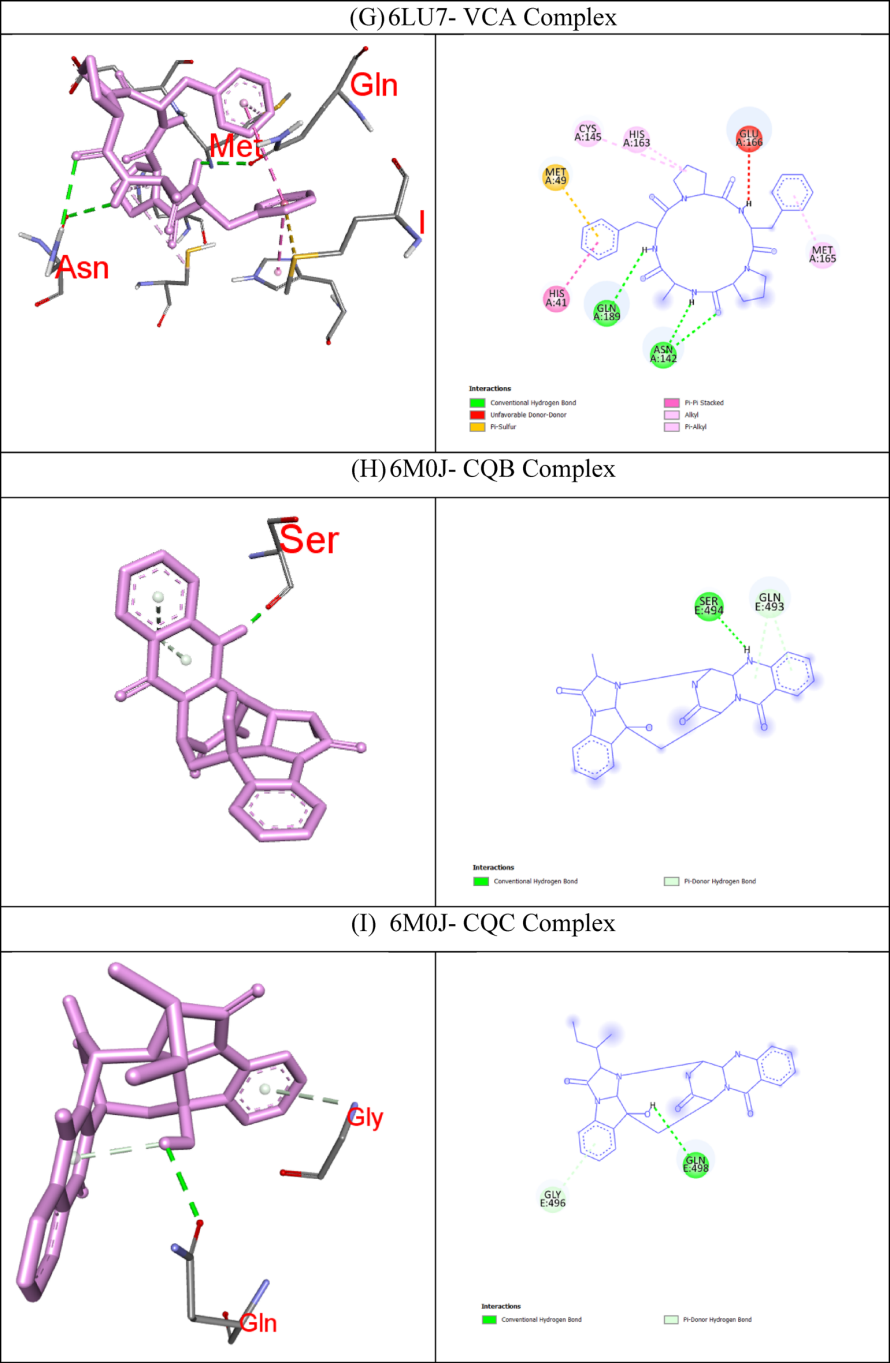

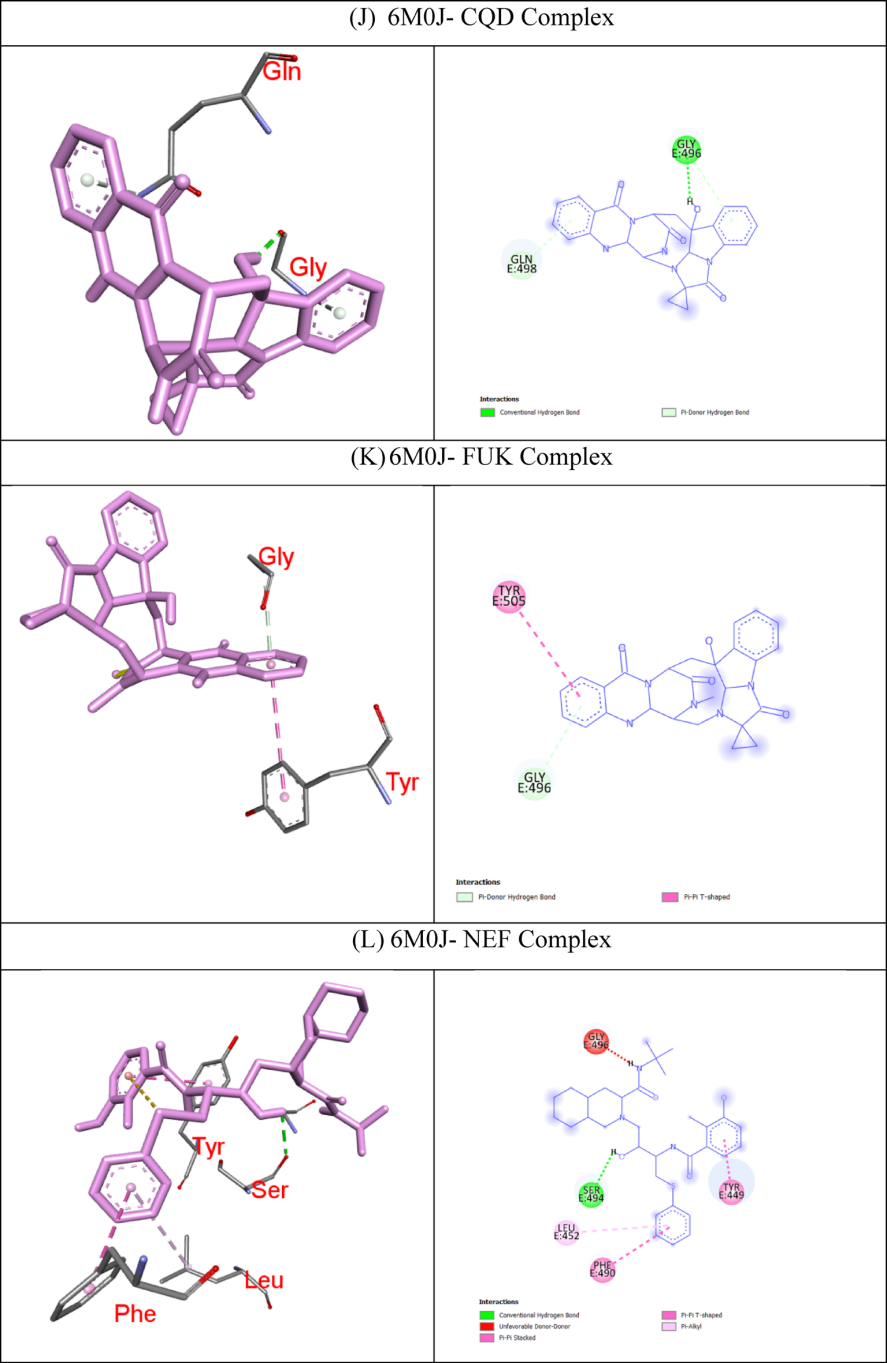

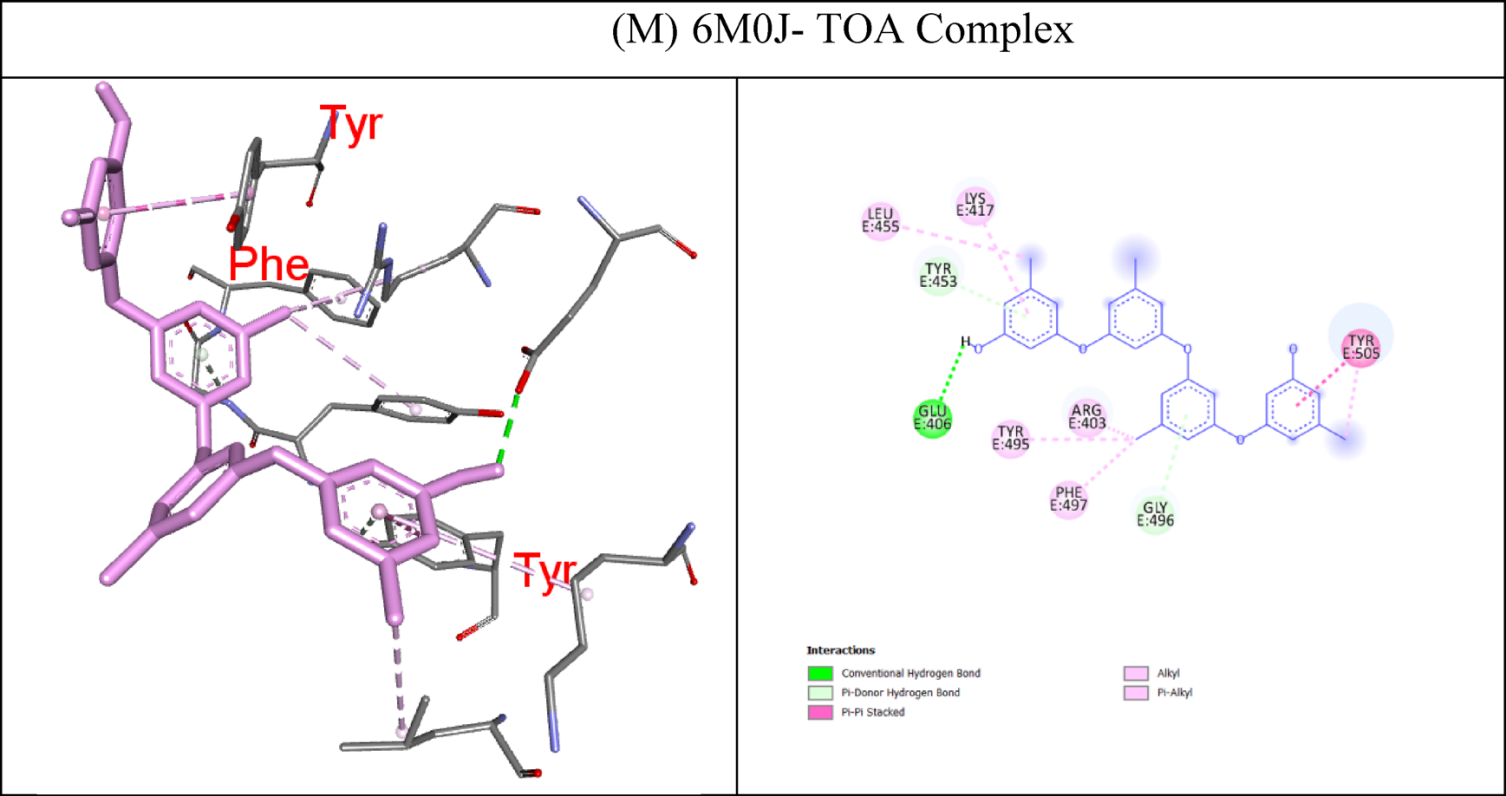
Binding modes (3D and 2D) of the best docked MNPs from soft coral-Derived microbes against SARS-CoV-2 Mpro and Spike protein. **(A)** 6LU7- CQB Complex, **(B)** 6LU7- CQD Complex, **(C)** 6LU7- FUK Complex, **(D)** 6LU7-REM Complex, **(E)** 6LU7- Native Ligand (N3) Complex, **(F)** 6LU7- TOA Complex, **(G)** 6LU7- VCA Complex, **(H)** 6M0J- CQB Complex, **(I)** 6M0J- CQC Complex, **(J)** 6M0J- CQD Complex, **(K)** 6M0J- FUK Complex, **(L)** 6M0J- NEF Complex, and 6M0J- TOA Complex.

In molecular docking, the key components for predicting potential hit molecules are based on the number of primary hydrogen-bond (H-bond) interactions and hydrophobic contacts. Both the H-bonding and hydrophobic interactions are crucial in facilitating significant ligand binding at the active-site residues of the receptor in docked complexes^31,32^. The intermolecular bonded interactions of only the top-docked complexes are listed in the Table 3 and their 2D and 3D structures are illustrated in Fig. 3. The selection of top docked complexes was also based on number of non-bonded interactions such as hydrophobic, electrostatic, and other secondary weak H-bond interactions. With 6LU7, Fumiquinazoline K formed three H-bonds and two hydrophobic interactions, Cottoquinazoline B formed four H-bonds and two hydrophobic contacts, Tetraorcinol A formed three H-bonds and five hydrophobic interactions, Cottoquinazoline D formed 3 H-bonds and 6 hydrophobic interactions, Versicoloritide A formed 3 hydrogen bond interactions and 4 hydrophobic interactions.

With 6M0J, Cottoquinazoline B formed three H-bond interactions, Cottoquinazoline C formed two H-bond interactions, Cottoquinazoline D formed three H-bond interactions, Fumiquinazoline K formed one H-bond interaction and one hydrophobic interaction, Nelfinavir showed one hydrogen bond and three hydrophobic interactions, Tetraorcinol A formed three H-bonds and eight hydrophobic interactions. As mentioned in Table 3, Native ligand (N3) showed a maximum of ten H-bond interactions and only one hydrophobic contact (THR25) towards 6LU7, while Remdesivir showed only one H-bond five hydrophobic contacts (HIS41, LEU27, CYS145, HIS41, MET49)towards 6LU7.

### Molecular docking results of MNPs from soft coral-derived microbes against the SARS-CoV-2 VOCs

It has been reported that several amino acids such as Q493, Y505, Q498, N501, T500, N487, Y449, F486, K417, Y489, F456, Y495, and L455 have been identified as hotspots in the RBD of SARS-CoV-2. The mutations in S-protein, especially in the RBD are critically involved in the evolution of diverse spike variants with variable virulence capacity. Five key mutations in the RBD of the SARS-CoV-2 S-protein include (1) Asparagine (ASN) to Tyrosine (TYR) at position 501 (N501Y), (2) Glutamic Acid (GLU) to Lysine (LYS) or Glutamine (GLN) at position 484 (E484K/Q), (3) Lysine (LYS) to Asparagine (ASN) or Threonine (THR) at position 417 (K417N/T), (4) Leucine (LEU) to Arginine (ARG) at position 452 (L452R), and (5) Threonine (THR) to Lysine (LYS) at position 478 (T478K), have significantly contributed to the biological impact of the virus. Therefore, we also performed molecular docking for test compounds against SARS-CoV-2 variants of concern (VOCs) such as Alpha (U.K, B.1.1.7) (PDB ID:7LWS), Beta (South African, B.1.351) (PDB ID: 7LYK), Delta (India, B.1.617.2) (PDB ID: 7V8B), and Omicron variants (PDB ID: 7T9J). Binding energies for all tested compounds against both Mpro and the RBD of WT and different VOCs are given in the supplementary table S1 (Excel file), while only best docked compounds were shown in Fig. 2. The 2D and 3D binding modes of the top five best docked MNPs against the RBD of S-protein from VOCs are provided in the supplementary Fig. 1.

The key amino acid residues that were involved in the interaction of RBD of S-protein of Alpha variant (7LWS) with the best docked compounds are shown in Table 4. As shown in Supplementary Fig. 1, Versicoloritide A formed 3 H-bonds (TYR453, TYR501, TYR453) and 5 hydrophobic interactions (TYR505, ARG403, TYR453, TYR495, TYR505), whereas Versicoloritide C formed 4 H-bonds (TYR453, TYR501, TYR495, TYR453) and 2 hydrophobic contacts (TYR505, TYR453). Alterporriol O interacted with the RBD of Alpha variant via 8 H-bonds and 3 hydrophobic interactions. The key amino acids involved in H-bond formation include TYR449, TYR449, SER494, TYR501, TYR495, TYR495, GLN493, and GLN493, while the amino acids involved in hydrophobic interactions include TYR501, TYR501, TYR505. On the contrary, Alterporriol Q formed only three H-bonds (TYR449, PHE490, LEU492) but formed ten hydrophobic contacts (GLU484, GLU484, GLU484, LEU452, TYR489, TYR489, PHE490, PHE490, LEU452, PHE490). Fumiquinazoline K formed 3 H-bonds (LEU492, GLN493, LEU492) and 3 hydrophobic contacts (GLU484, PHE490, LEU452).

MNPs such as Cottoquinazoline B and D, Fumiquinazoline K, Tetraorcinol A, and Versicoloritide A, constitute the top 5 best docked compounds. The key amino acid residues that were involved in the interaction of the RBD of S-Protein of the beta variant (7LYK) with the best-docked compounds are shown in Table 4. Tetraorcinol A formed 2 H-bonds (THR500 and TYR453) and 8 hydrophobic contacts (TYR505, TYR505, ARG403, TYR495, PHE497, TYR501, TYR505, TYR505). Whereas Versicoloritide A formed 2 H-bonds (TYR495 and TYR501) but only one hydrophobic contact (TYR505). Both Cottoquinazoline B and D formed 3 H-bonds each with amino acids such as SER494, TYR449, and GLN493. However, Cottoquinazoline D formed 4 hydrophobic interactions with TYR449, LEU452, PHE490, and LYS484 and Cottoquinazoline B formed 3 hydrophobic contacts with TYR449, TYR489, LYS484. Despite being the top docked compound with the lowest binding energy of −7.2 Kcal/mol, Fumiquinazoline K formed only one H-bond with TYR501 of the S-Protein of the beta variant.

Alterporriol Q, Cottoquinazoline B, D, Fumiquinazoline K, and Tetraorcinol A constitute the top 5 best docked compounds against the RBD of the S-protein of the Omicron variant (7T9J). As shown in Table 4, Cottoquinazoline B and D formed 3 (ARG403, SER494, SER496) and 4 H-bonds (ARG403, SER494, SER496, SER496), respectively but 1 (HIS505) and 3 hydrophobic contacts (ARG403, TYR495, PHE497), respectively. Fumiquinazoline K formed 2 H-bonds (ARG403 and SER494) but 7 hydrophobic contacts (ARG493, TYR453, ARG493, ARG403, TYR495, ARG493, ARG493). Tetraorcinol A formed 5 H-bonds (SER494, SER496, TYR495, TYR453, SER496) and 7 hydrophobic contacts (HIS505, ARG493, TYR449, TYR453, TYR501, HIS505, ARG493). Alterporriol Q formed only 2 H-bonds (SER349 and ARG493) and formed 9 hydrophobic contacts (LEU452, LEU452, LEU452, PHE490, PHE490, PHE490, TYR489, ARG493, LEU452).

Compounds such as Fumiquinazoline K, Cottoquinazoline B and D, Tetraorcinol A, Versicoloritide B constitute the top 5 best docked compounds against the RBD of the S-protein of the Delta variant (7V8B). The key amino acid residues that were involved in the interaction of the RBD Omicron variant with the best-docked compounds are shown in Table 4. Despite the highest binding affinity, Fumiquinazoline K showed only one H-bond (GLY496) and three hydrophobic contacts (TYR505, GLY496, PHE497). Cottoquinazoline D formed 2 H-bonds (ASN501 and GLY496) and only one hydrophobic contact (TYR505), whereas Cottoquinazoline B formed 2 H-bonds (GLY496 and GLN498) and 4 hydrophobic contacts (TYR505, TYR505, GLY496, PHE497), Versicoloritide B formed only 2 H-bonds (GLY496 and ASN501) and only one hydrophobic contact (TYR505). Tetraorcinol A formed 5 H-bonds (TYR505, ARG403, PHE497, TYR453, and TYR453) and 8 hydrophobic contacts (LEU455, TYR453, TYR505, LYS417, LEU455, TYR453, PHE456, TYR505).

Alterporriol Q, Tetraorcinol A, Versicoloritide A, Alterporriol P, and Fumiquinazoline K constitute the top 5 best docked compounds against the RBD of the S-protein of the Gamma variant (7V82). The key amino acid interactions between them are shown in Table 4. Alterporriol Q formed only one H-bond (TYR351) but 7 hydrophobic contacts (LEU452, LEU452, PHE490, PHE490, PHE490, LEU452, LEU452). Tetraorcinol A formed 3 H-bonds (GLN493, LYS484, PHE490) and 11 hydrophobic contacts (TYR489, PHE490, ILE472, LYS484, LYS484, PHE456, TYR489, PHE490, PHE490, ILE472, LYS484). Versicoloritide A, Alterporriol P, and Fumiquinazoline K formed one (GLN493), two (TYR351 and TYR351), and one (TYR501) H-bond and four (TYR505, TYR449, TYR501, TYR505), six (LEU452, LEU452, PHE490, PHE490, PHE490, LEU452), four (TYR505, TYR505, TYR501, TYR501) hydrophobic contacts, respectively.

**Table 2:** Physicochemical properties of best docked MNPs from soft coral-derived microbes.

Ligand name	Molecular formula	Molecular Weight (< 500 Da)	No. of HBA (≤ 12)	No. of HBD (≤ 7)	Mol LogP^c^ (≤ 5)	Mol LogS^d^ (0 to −6)	MolPSA (Å^2^) (90–140Å)	No. of stereo centers (0–5)
*Alterporriol P*	*C*_*32*_ *H*_*26*_ *O*_*12*_	*602.5*	*12*	*6*	*2.95*	*−2.99*	*161.07*	*3*
*Alterporriol Q*	*C*_*32*_ *H*_*22*_ *O*_*10*_	*566.5*	*10*	*4*	*5.90*	*−5.25*	*131.08*	*0*
*Alterporriol O*	*C*_*32*_ *H*_*30*_ *O*_*14*_	*638.6*	*14*	*8*	*−0.22*	*−1.27*	*192.89*	*8*
Aspergillipeptide C	C_26_H_36_N_4_O_8_	532.25	8	6	0.79	−1.71	155.89	5
Cottoquinazoline B	C_23_H_19_N_5_O_4_	429.4	6	2	0.28	−2.03	82.28	5
Cottoquinazoline C	C_26_H_25_N_5_O_4_	471.5	6	2	1.55	−2.26	82.69	6
Cottoquinazoline D	C_24_H_19_N_5_O_4_	441.4	6	2	0.39	−2.03	82.73	4
Fumiquinazoline K	C_26_H_23_N_5_O_4_	469.5	6	1	0.44	−2.01	73.84	4
Aqabamycin c	C_16_H_10_N_2_O_5_	310.26	5	2	1.42	−2.15	93.24	0
Versicoloritide-A	C_31_ H_37_N_5_O_5_	559.28	5	3	1.15	−1.68	110.14	5
Versicoloritide-C	C_31_H_37_N_5_O_6_	575.27	6	4	0.46	−1.42	127.86	5
Tetraorcinol A	C_28_H_26_O_5_	442.5	5	2	7.26	−5.87	55.72	0
Triterpenoid	C_30_H_48_O_7_S	552.8	7	3	2.60	−2.95	96.68	9
Aqabamycin G	C1_8_H_11_N_3_O_5_	349.3	5	3	1.77	−2.47	103.11	0

**Table 3:** The intermolecular interaction of best docked complexes of Mpro (6LU7) and WT Spike RBD (6M0J).

**PDB ID**	**Native/test Ligand (Binding Energy)**	**H-bond**	**H-bond length(Å)**	**No of H- bonds**	**Hydrophobic Contacts**
		**Amino acid residues involved in the bond formation**			
6LU7	Fumiquinazoline K (FUK) (−9.4 Kcal/mol)	GLN189	2.60	3	HIS41, MET165
		GLN189	3.53		
		ASN142	2.89		
	CottoquinazolineB (CQB) (−9.3 Kcal/mol)	HIS41	2.77	4	CYS145, MET165
		HIS163	2.28		
		MET165	3.29		
		ASN142	3.24		
	Tetraorcinol (TOA) (−9.3 Kcal/mol)	GLY143	2.40	3	LEU27, HIS41, HIS41, MET49, CYS145
		ARG188	1.98		
		ASN142	3.50		
	Cottoquinazoline D (CQD) (−9.2 Kcal/mol)	GLN189	2.22	3	HIS41, HIS41, LEU27, HIS41, CYS145, MET165
		ASN142	3.22		
		HIS41	2.63		
	Versicoloritide A (VCA) (−9.2 Kcal/mol)	ASN142	2.98	3	HIS41, CYS145, HIS163, MET165
		ASN142	2.70		
		GLN189	2.73		
	Native Ligand (N3) (−7.6 Kcal/mol)	HIS41	2.81	10	THR25
		GL143	2.25		
		SER144	2.74		
		HIS163	2.40		
		GLU166	2.12		
		GLN189	2.16		
		GLN189	2.39		
		GLU166	2.79		
		GLU166	3.65		
		THR25	3.73		
	Remdesivir (REM) (−7.9 Kcal/mol)	GLU406	2.67	1	HIS41, LEU27, CYS145, HIS41, MET149
6M0J	Cottoquinazoline B (CQB) (−7.9 Kcal/mol)	SER494	1.85	3	-
		GLN493	3.32		
		GLN493	3.98		
	Cottoquinazoline C (CQC) (−7.5 Kcal/mol)	GLN498	2.67	2	-
		GLY496	3.79		
	Cottoquinazoline D (CQD) (−8.0 Kcal/mol)	GLY496	2.06	3	-
		GLY496	3.78		
		GLN498	3.63		
	Fumiquinazoline K (FUK) (−7.6 Kcal/mol)	GLY496	3.77	1	TYR505
	Nelfinavir (NER) (−7.1 Kcal/mol)	SER494	2.38	1	TYR449, PHE490, LEU452
	Tetraorcinol (TOA) (−7.6 Kcal/mol)	GLU406	2.67	3	TYR453, TYR505, ARG403, LEU455, TYR495, PHE497, TYR505, LYS417
		TYR453	3.86		
		GLY496	3.98		

CQB -CottoquinazolineB; CQC – CottoquinazolineC; CQD-CottoquinazolineD; FUK-Fumiquinazoline K; NEF-Nelfinavir; REM-Remdesivir; TOA-Tetraorcinol A; VCA-Versicoloritide A.

**Table 4:** The intermolecular interaction of best docked complexes of MNPs from soft coral-derived microbes against RBD of Spike variants of concern (VOCs) of SARS-CoV-2.

**PDB** **ID**	**Native/test** **Ligand** **(Binding** **energy)**	**H-bond**	**H-bond** **length** **(Å)**	**No of** **H-bonds**	**Hydrophobics contacts**
		**Amino acid residues involved in the bond formation**			
7LWS (Alpha)	VCA (−8.4 Kcal/mol)	TYR453	3.25	3	TYR505, ARG403, TYR453, TYR495, TYR505
		TYR501	3.33		
		TYR453	3.13		
	APO (−8.3 Kcal/mol)	TYR449	2.92	8	TYR501, TYR501, TYR505
		TYR449	3.09		
		SER494	1.93		
		TYR501	2.43		
		TYR495	3.35		
		TYR495	3.35		
		GLN493	4.0		
		GLN493	3.58		
	FUK (−8.1 Kcal/mol)	LEU492	2.25	3	GLU484, PHE490, LEU452
		GLN493	2.59		
		LEU492	3.67		
	VCC (−8.1 Kcal/mol)	TYR453	3.33	4	TYR505, TYR453
		TYR501	3.33		
		TYR495	2.57		
		TYR453	3.09		
	APQ (−7.9 Kcal/mol)	TYR449	2.59	3	GLU484, GLU484, GLU484, LEU452, TYR489, TYR489, PHE490, PHE490, LEU452, PHE490
		PHE490	2.12		
		LEU492	2.33		
7LYK (Beta)	TOA (−7.5 Kcal/mol)	THR500	3.28	2	TYR505, TYR505, ARG403, TYR495, PHE497, TYR501, TYR505, TYR505
		TYR453	4.08		
	VCA (−7.5 Kcal/mol)	TYR495	2.37	2	TYR505
		TYR501	2.09		
	CQD (−7.4 Kcal/mol)	SER494	3.26	3	TYR449, LEU452, PHE490, LYS484
		TYR449	3.78		
		GLN493	3.88		
	CQB (−7.3 Kcal/mol)	SER494	3.26	3	TYR449, TYR489, LYS484
		TYR449	4.01		
		GLN493	3.94		
	FUK (−7.2 Kcal/mol)	TYR501	3.54	1	-
7T9J (Omicron)	CQB (−8.0 Kcal/mol)	ARG403	2.89	3	HIS505
		SER494	2.92		
		SER496	3.55		
	CQD (−7.9 Kcal/mol)	ARG403	3.18	4	ARG403, TYR495, PHE497
		SER494	2.68		
		SER496	2.67		
		SER496	4.07		
	FUK (−7.9 Kcal/mol)	ARG403	3.16	2	ARG493, TYR453, ARG493, ARG403, TYR495, ARG493, ARG493
		SER494	1.93		
	TOA (−7.3 Kcal/mol)	SER494	3.37	5	HIS505, ARG493, TYR449, TYR453, TYR501, HIS505, ARG493
		SER496	2.48		
		TYR495	3.73		
		TYR453	4.14		
		SER496	3.51		
	APQ (−7.0 Kcal/mol)	SER349	2.88	2	LEU452, LEU452, LEU452, PHE490, PHE490, PHE490, TYR489, ARG493, LEU452
		ARG493	3.27		
7V8B (Delta)	FUK (−8.3 Kcal/mol)	GLY496	3.22	1	TYR505, GLY496, PHE497
	CQD (−7.5 Kcal/mol)	ASN501	3.05	2	TYR505
		GLY496	1.69		
	VCB (−7.6 Kcal/mol)	GLY496	2.54	2	TYR505
		ASN501	3.44		
	TOA (−7.5 Kcal/mol)	TYR505	1.89	5	LEU455, TYR453, TYR505, LYS417, LEU455, TYR453, PHE456, TYR505
		ARG403	3.55		
		PHE497	3.46		
		TYR453	4.01		
		TYR453	3.73		
	CQB (−7.9 Kcal/mol)	GLY496	2.87	2	TYR505, TYR505, GLY496, PHE497
		GLN498	3.78		
7V82 (Gamma)	APQ (−7.6 Kcal/mol)	TYR351	3.29	1	LEU452, LEU452, PHE490, PHE490, PHE490, LEU452, LEU452
	TOA (−7.5 Kcal/mol)	GLN493	3.20	3	TYR489, PHE490, ILE472, LYS484, LYS484, PHE456, TYR489, PHE490, PHE490, ILE472, LYS484
		LYS484	3.50		
		PHE490	3.90		
	VCA (−7.4 Kcal/mol)	GLN493	3.52	1	TYR505, TYR449, TYR501, TYR505
	APP (−7.2 Kcal/mol)	TYR351	3.29	2	LEU452, LEU452, PHE490, PHE490, PHE490, LEU452
		TYR351	2.90		
	FUK (−7.1 Kcal/mol)	TYR501	2.94	1	TYR505, TYR505, TYR501, TYR501

VCA: Versicoloritide A, VCB: Versicoloritide B, FUK-Fumiquinazoline K, VCC: Versicoloritide C, APO: Alterporriol O, APQ: Alterporriol Q, TOA: Tetraorcinol A, CQD: Cottoquinazoline D, CQB: Cottoquinazoline B.

### Analysis of ADMET properties of coral associated microbial natural compounds

Predicting the absorption, distribution, metabolism, excretion and toxicity (ADMET) parameters in drug development is an important step to prevent drug failure in clinical stages. The best docked compounds were predicted for drug-likeness and ADMET parameters with the help of a web-based server ADMETlab 2.0^33^. A molecule failing to meet ADMET properties may indicate that the molecule may not be properly absorbed or metabolized, and may be toxic too, thus preventing that drug from being used clinically in the future^27,34,35^. Most of the best docked compounds obeyed Lipinski’s rule of five and are predicted as non-carcinogenic.

From the ADMET data (Table 5), it can be predicted that compounds such as Versicoloritide-A, -B, and -C exhibit strong P-glycoprotein (Pgp) inhibition (0.982, 0.976, 0.936) and high human intestinal absorption (HIA: 0.808–0.989), suggesting potential as bioavailability enhancers, though they may inhibit CYP3A4 (0.873–0.93) and Versicoloritide-C violates Lipinski’s rule. Cottoquinazolines (B/C/D) show favorable drug-likeness (Lipinski-accepted), moderate Pgp inhibition (0.101–0.723), and low toxicity (Ames: 0.019–0.024), making them promising candidates. Aspergillipeptide C is a likely Pgp substrate (0.987) with poor absorption (HIA: 0.028) and a PAINS alert, limiting its utility, while Alterporriols (Q/P) also trigger PAINS alerts despite moderate Pgp inhibition. Fumiquinazoline-K has good oral bioavailability potential (0.919) but raises carcinogenicity concerns (0.438). Triterpenoid shows negligible Pgp effects (inh: 0.013) but high carcinogenicity risk (0.583). Compounds like Tetraorcinol A (Lipinski-accepted, low HIA: 0.036) and Versicoloritides may require formulation optimization for delivery, whereas Cottoquinazolines and Fumiquinazoline-K (despite risks) emerge as more viable leads.

**Table 5:** ADMET properties of top hit coral associated microbial natural compounds.

Pgp-inh	Aspergillipeptide C	Alterporriol Q	Alterporriol *P*	Cottoquinazoline-B	Cottoquinazoline-C	Cottoquinazoline-D	Fumiquinazoline-K	Triterpenoid	Versicoloritide-A	Versicoloritide-B	Versicoloritide-C	Tetraorcinol A
	0.638	0.855	0.831	0.101	0.723	0.304	0.831	0.013	0.982	0.976	0.936	0.666
**Pgp-sub**	0.987	0.017	0.949	0.009	0.017	0.005	0.004	0	0.016	0.013	0.014	0.001
**HIA**	0.028	0.827	0.95	0.509	0.453	0.093	0.136	0.781	0.808	0.91	0.989	0.036
**Caco-2**	−5.174	−6.108	−6.359	−5.553	−5.371	−5.377	−5.182	−5.384	−5.727	−5.762	−6.111	−5.678
**BBB**	0.734	0.001	0.001	0.221	0.183	0.647	0.55	0.17	0.007	0.011	0.017	0.027
**CYP3A4-inh**	0.645	0.178	0.074	0.085	0.539	0.545	0.861	0.04	0.93	0.928	0.873	0.302
**Ames**	0.013	0.29	0.567	0.02	0.019	0.024	0.031	0.034	0.009	0.009	0.008	0.061
**ROA**	0.08	0.088	0.064	0.375	0.709	0.61	0.919	0.123	0.985	0.972	0.991	0.046
**Carcinogenicity**	0.037	0.352	0.022	0.057	0.074	0.065	0.438	0.583	0.014	0.012	0.024	0.233
**PAINS**	1	1	1	0	0	0	0	0	0	0	0	0
**Lipinski**	Rejected	Rejected	Rejected	Accepted	Accepted	Accepted	Accepted	Accepted	Accepted	Accepted	Rejected	Accepted

## MD simulation results

### Stability of Cottoquinazoline B (CQB), native ligand (N3), and Remdesivir (REM) with the Mpro

**Fig. 4:**
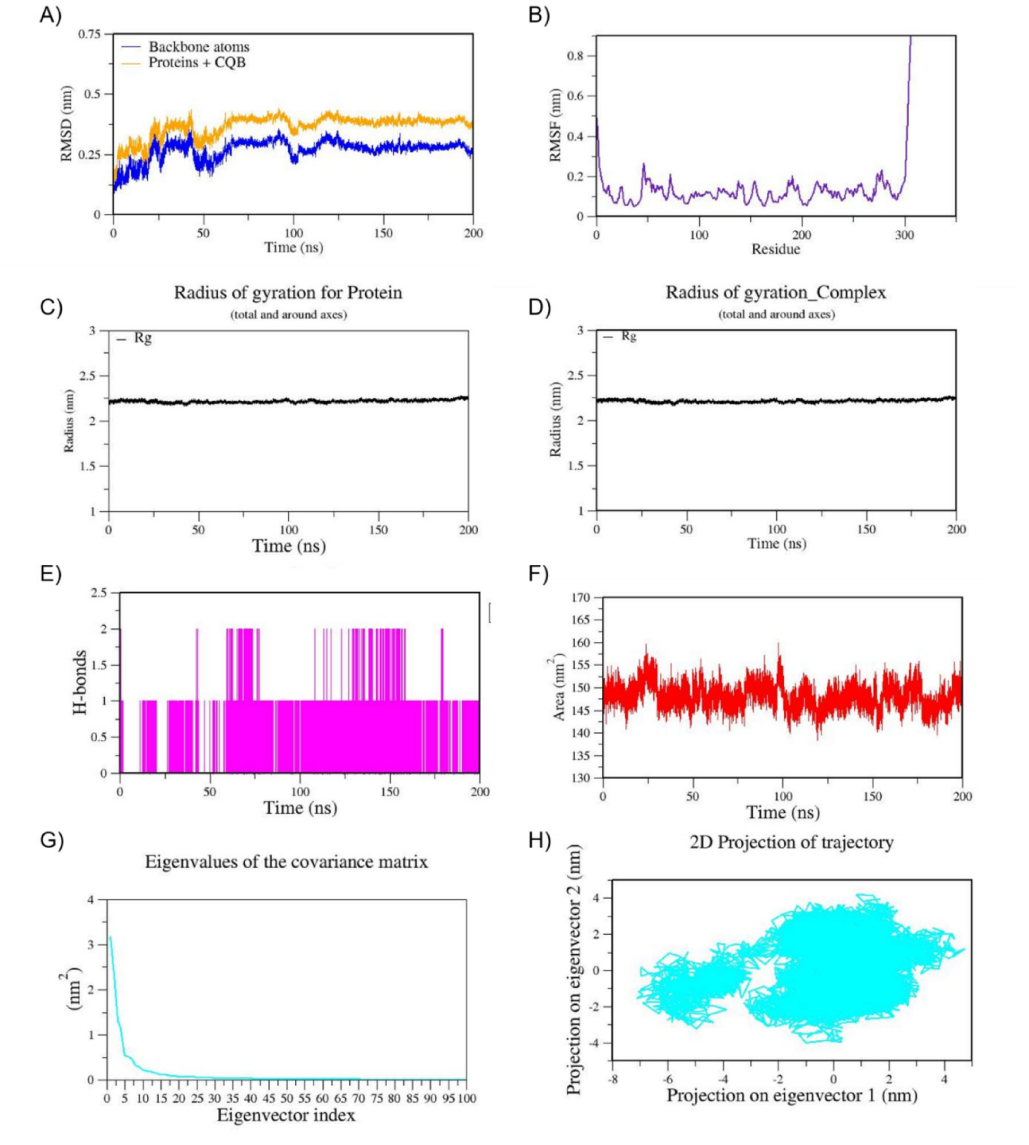
MD trajectories of 6LU7_CQB during 200 ns MD simulation. **(A)** RMSD **(B)** RMSF **(C)** Radius of gyration of Protein **(D)** Radius of gyration of Complex **(E)** H-Bonds **(F)** SASA **(G)** Eigen values f the covariance matrix and **(H)** 2D Projection of trajectory.

Based on the docking results we have chosen Cottoquinazoline B which has shown much higher binding affinity (−9.3 kcal/mol) against Mpro when compared to the known approved drug remdesivir (ΔG=−7.9 kcal/mol). Interestingly, intermolecular interactions formed by Cottoquinazoline B in docking are highly conserved with key catalytic residues. The non-bonded contacts and H-bonding interactions are much stronger compared to other best docked poses. All-atom MD simulation of the Mpro-cottoquinazoline B- (CQB-6LU7) complex for 200 ns in explicit solvent reveals stable dynamics throughout the simulation period. This system has reached equilibrium up to 100 ns thereafter the overall complex is well stabilized as shown in the RMSD plot (Fig. 4). The backbone RMSD shown in Fig. 4A represents a least deviation during the simulation i.e., 3.0 nm and 3.5 nm, whereas the RMSD value for the protein-drug complex is relatively higher due to side chain fluctuations (i.e. 3.5 nm to 4.0 nm) yet still within a stable range. This indicates that there are no major conformational changes during the simulation in this complex. When considering the RMSF, reflecting additional mobility due to ligand dynamics, the per residue fluctuation was monitored by measuring Root Mean Square Fluctuation (RMSF) values plotted in Fig. 4B. It represents the stable interactions at the binding pocket region < 0.2 nm and other folded regions; however, loop regions, both N- and C-terminals show higher fluctuations due to their disordered nature. The stable binding of the ligand favors compact folding as revealed by the Radius of Gyration (Rg) (Fig. 4C and D), which remained relatively constant throughout the trajectory, that varies between 2.20 and 2.23 nm. Consistent with this observation, the Solvent Accessible Surface Area (SASA) values (Fig. 4F) fluctuated within 150–160 nm², indicating stable solvent exposure and no substantial conformational rearrangement that would alter the protein’s surface properties.

**Fig. 5:**
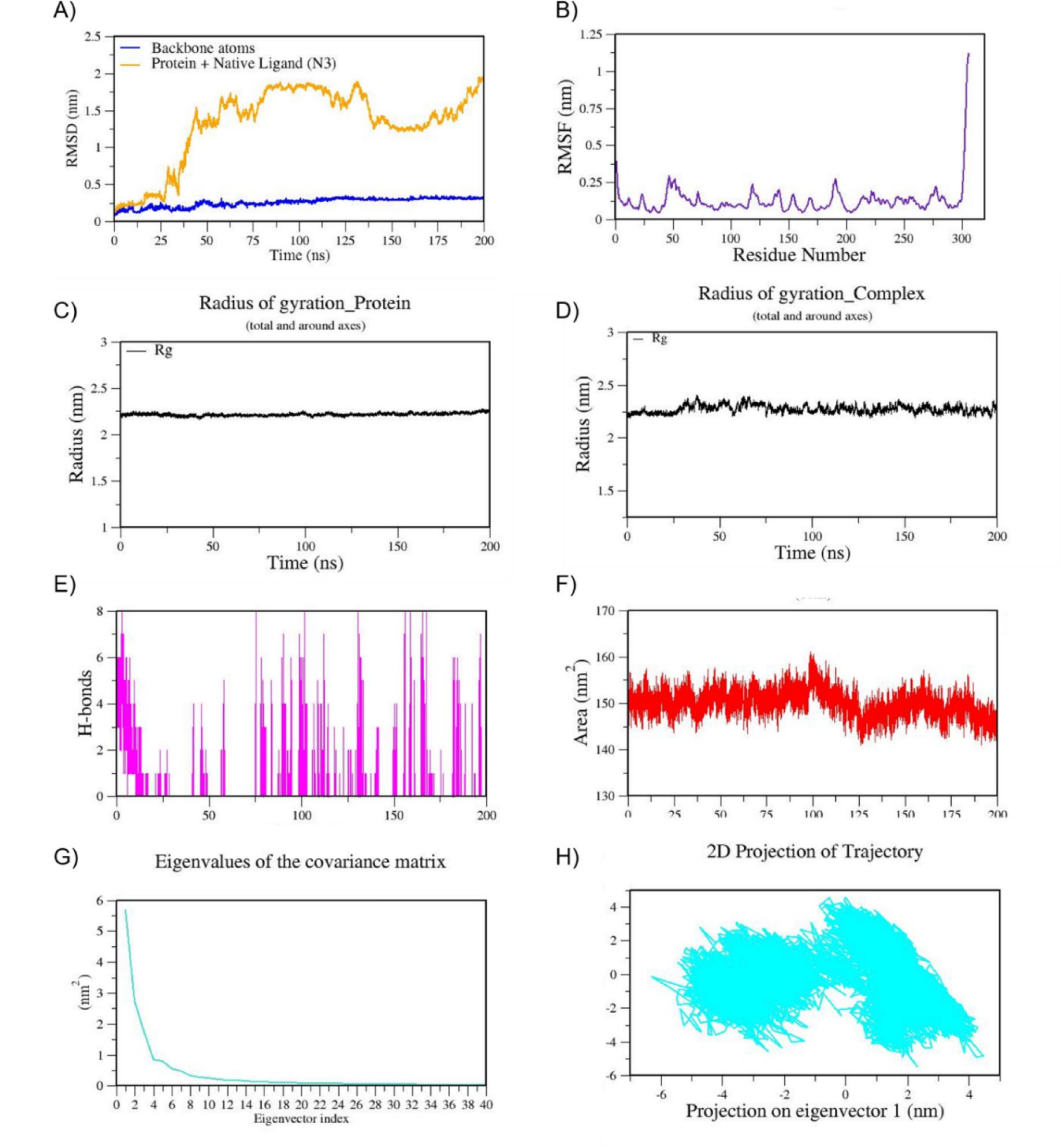
MD trajectories of 6LU7_N3 during 200 ns MD simulation. **(A)** RMSD **(B)** RMSF **(C)** Radius of gyration of Protein **(D)** Radius of gyration of Complex **(E)** H-Bonds **(F)** SASA **(G)** Eigen values f the covariance matrix and **(H)** 2D Projection of trajectory.

**Fig. 6:**
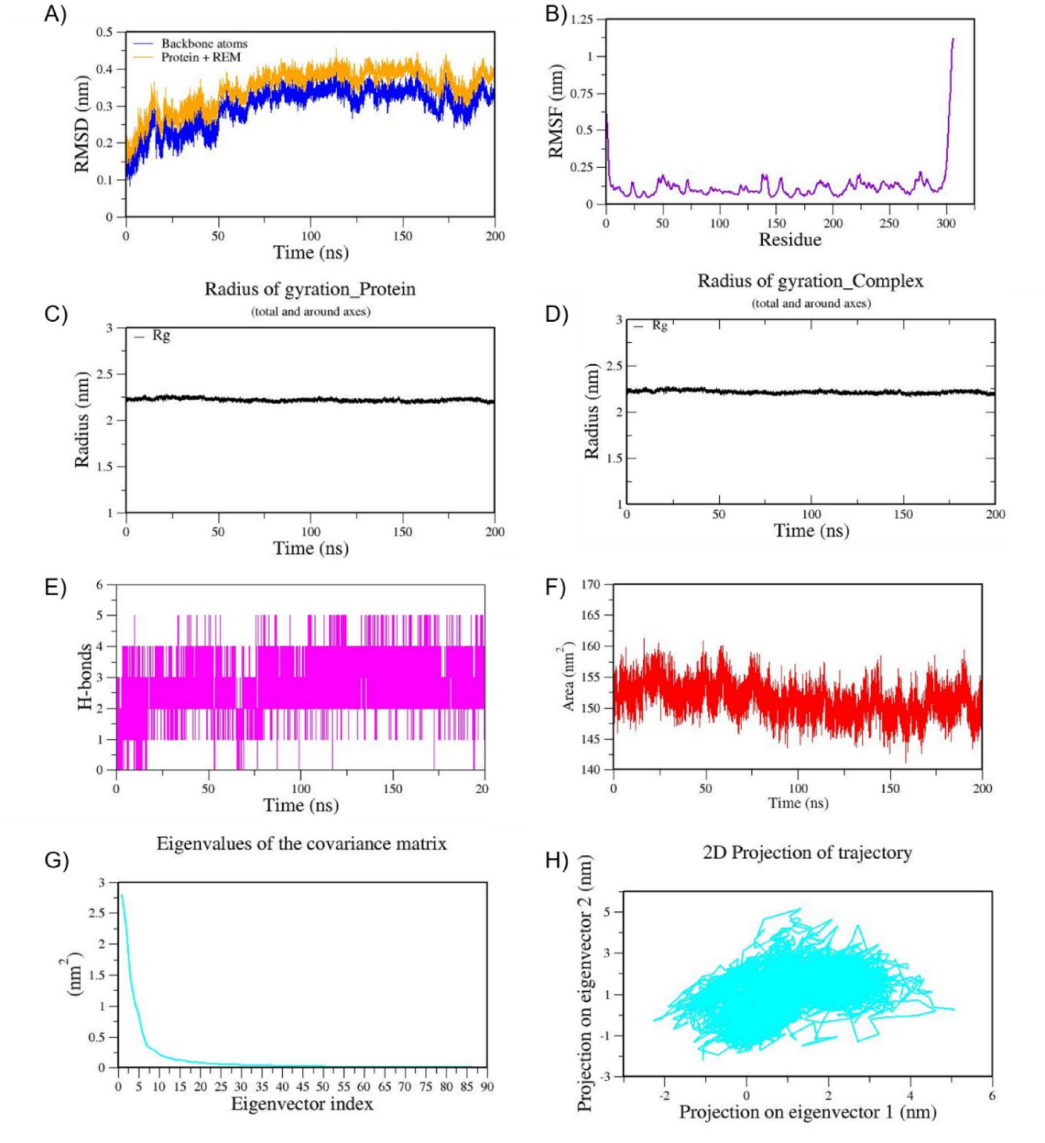
MD trajectories of 6LU7_REM during 200 ns MD simulation. **(A)** RMSD **(B)** RMSF **(C)** Radius of gyration of Protein **(D)** Radius of gyration of Complex **(E)** H-Bonds **(F)** SASA **(G)** Eigen values f the covariance matrix and **(H)** 2D Projection of trajectory.

The intermolecular non-bonded interactions during simulations were plotted including H-bonds and other hydrophobic contacts. This analysis indicates the formation of one consistent H-bond by CQB with the main protease; however, another H-bond formed after the equilibration period was not consistent (Fig. 4E). The trajectory analysis confirms the dominant role of other non-bonded contacts (hydrophobic or van der Waals forces) in stabilizing this complex, although the number of H-bonds was limited. The PCA analysis was performed to explore the conformational flexibility and overall stability during the MD simulation. It is observed that the first two eigenvectors capture the significant collective motion (Fig. 4G). As shown in Fig. 4H, the 2D projection of the MD trajectory results imply intermediate conformational stability of the CQB-Mpro complex due to a moderately dispersed cluster. The MD simulation trajectories of the native ligand_Mpro (N3-6LU7) and control drug remdesivir_Mpro (REM-6LU7) were represented in Figs. 5 and 6, respectively.

### Stability of Cottoquinazoline B (CQB) and nelfinavir (NEF) with the Spike receptor-binding domain (RBD)

**Fig. 7:**
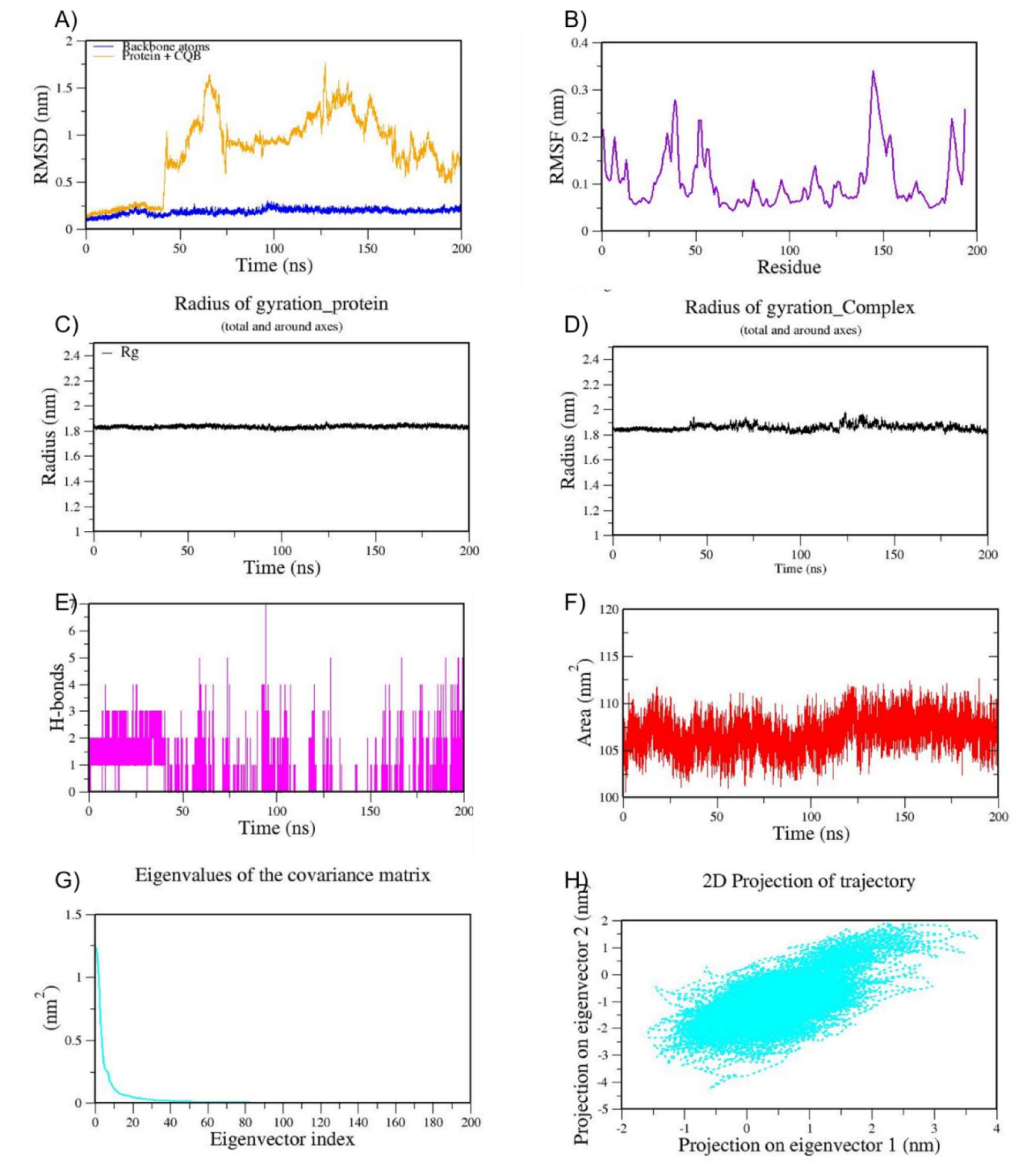
MD trajectories of highlighting the stability of cottoquinazoline B with spike receptor-binding domain (6M0J_CQB) during 200 ns MD simulation. **(A)** RMSD **(B)** RMSF **(C)** Radius of gyration of Protein **(D)** Radius of gyration of Complex **(E)** H-Bonds **(F)** SASA **(G)** Eigen values of the covariance matrix and **(H)** 2D Projection of trajectory.

**Fig. 8:**
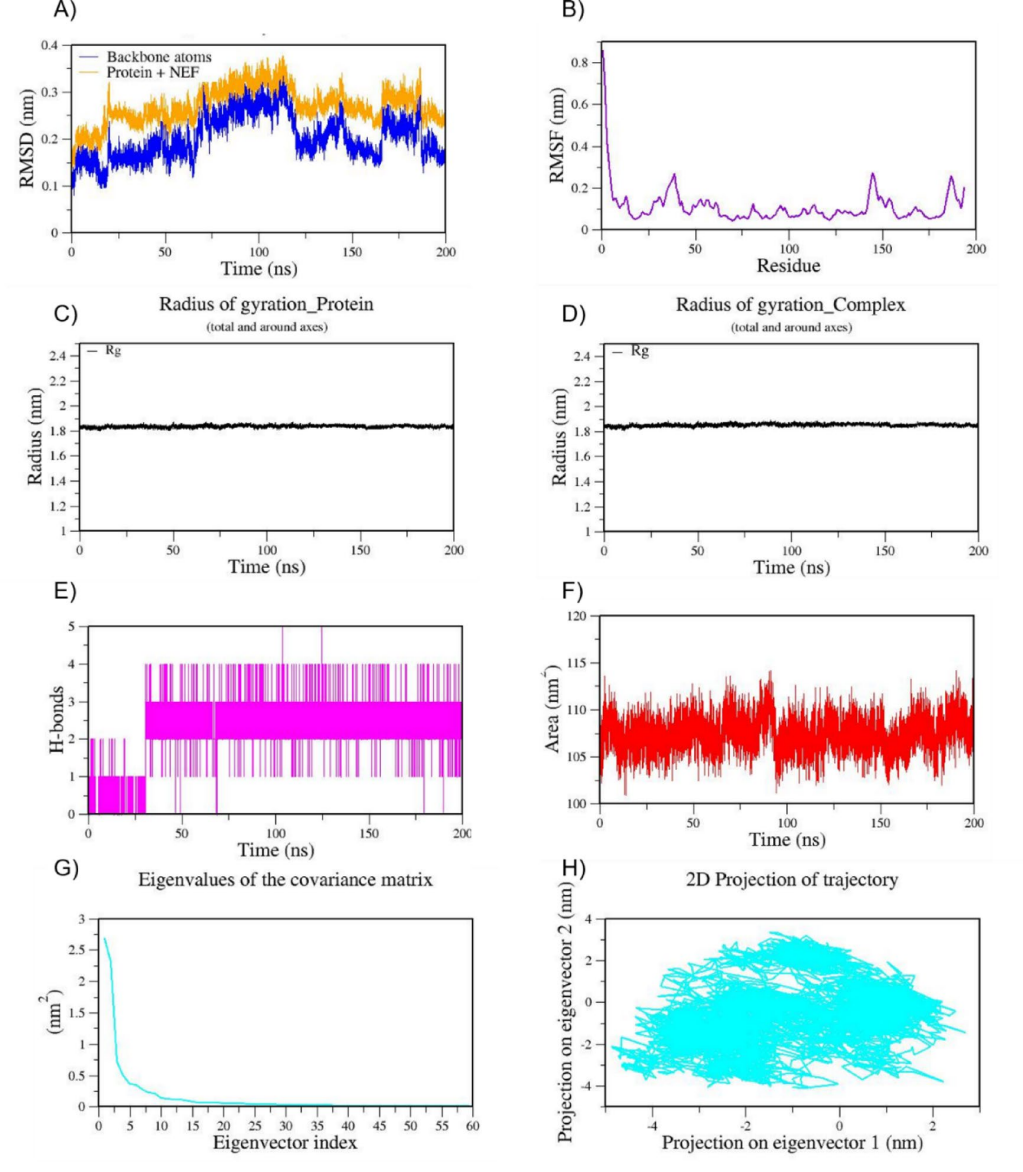
MD trajectories of highlighting the stability of nelfinavir (NEF) with spike receptor-binding domain (6M0J_NEF) during 200 ns MD simulation. **(A)** RMSD **(B)** RMSF **(C)** Radius of gyration of Protein **(D)** Radius of gyration of Complex **(E)** H-Bonds **(F)** SASA **(G)** Eigen values of the covariance matrix and **(H)** 2D Projection of trajectory.

The molecular dynamics simulation of the cottoquinazoline B–spike receptor-binding domain (RBD) (CQB_6M0J) complex over a 200 ns timescale reveals notable fluctuations in ligand stability with consistent structural integrity of the receptor (Fig. 7). The RMSD profile (Fig. 7A) for the protein backbone remains relatively stable throughout the trajectory, fluctuating around 0.25–0.30 nm, suggesting that the RBD maintains its global conformation. However, the RMSD of the protein–ligand complex (red line) shows a sharp increase after ~ 40 ns, rising above 1.5 nm, followed by a partial decline and re-stabilization around 0.9–1.0 nm after ~ 75 ns, indicating a significant rearrangement of the ligand within the binding pocket or potential repositioning. The RMSF plot (Fig. 7B) indicates moderate flexibility in specific loop regions and terminal residues, with fluctuations reaching up to 0.3–0.35 nm, particularly within the N-terminal (residues 1–40) and C-terminal regions, which are generally more solvent-exposed and dynamic. Central core residues of the RBD remain comparatively rigid (Fig. 7B), with RMSF values largely below 0.15 nm, indicating structural conservation in the binding interface. The Radius of Gyration (Rg) values (Fig. 7C and D) remain stable in the range of 1.80–1.86 nm, indicating that the RBD preserves a compact fold despite ligand-induced perturbations. Similarly, the Solvent Accessible Surface Area (SASA) (Fig. 7F) fluctuates minimally around 100–110 nm², reflecting a consistent exposure of the protein surface to the solvent, with no major folding or unfolding events. The hydrogen bond analysis reveals frequent and persistent interactions between Cottoquinazoline B and the RBD, with the number of hydrogen bonds fluctuating between one and six throughout the simulation (Fig. 7E). This indicates that, despite the observed ligand movement, key polar interactions are sustained, supporting dynamic yet recurring binding events. The PCA analysis of CQB-6M0J complexes shown in Figs. 7G and H suggests intermediate conformational stability of the CQB-spike protein complex due to a moderately dispersed cluster. The MD simulation trajectories of the control ligand nelfinavir with the spike receptor binding domain (NEF_6M0J) are shown in Fig. 8.

### Stability of best docked complexes of MNPs with RBDs of Spike VOCs

**Fig. 9:**
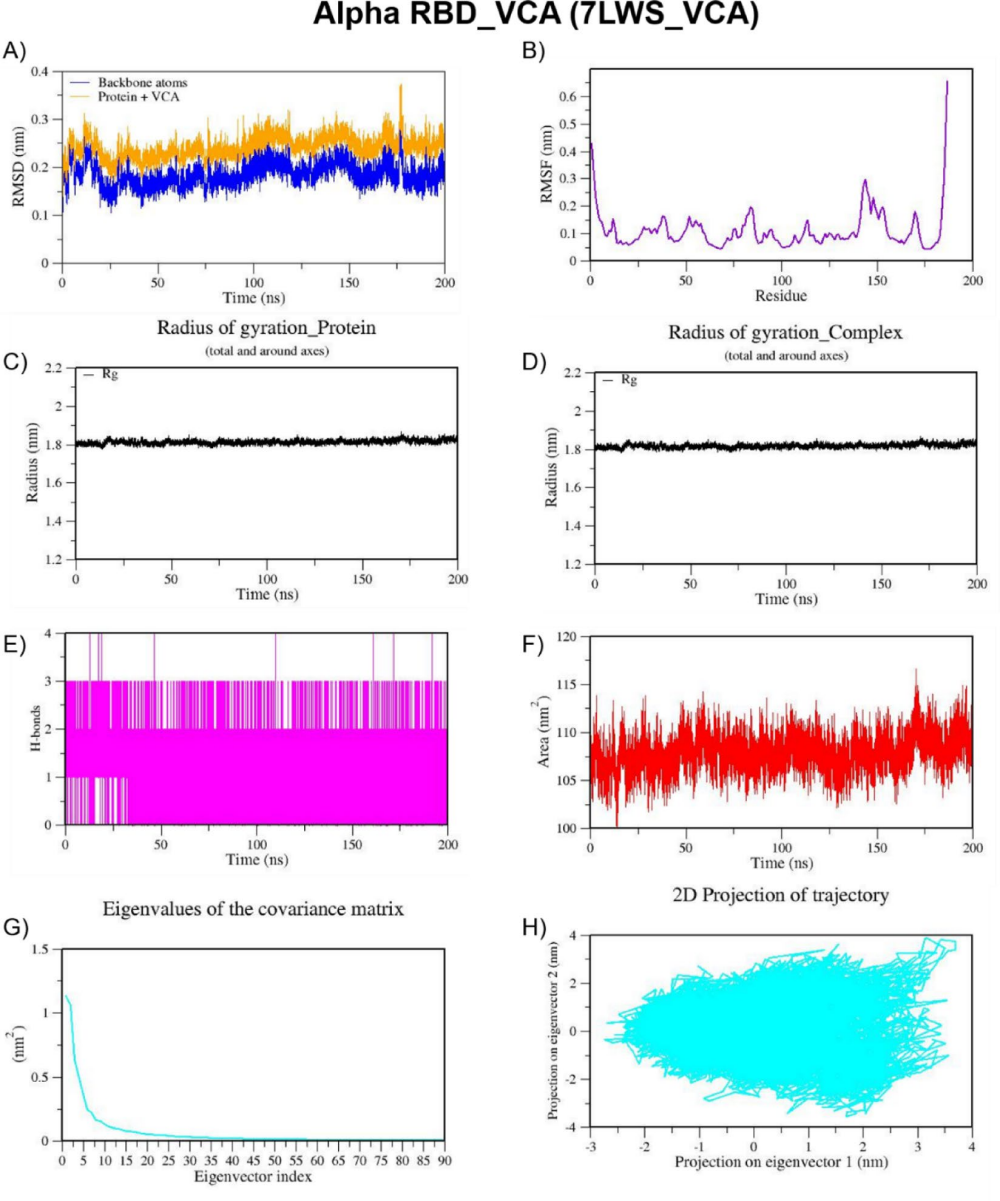
MD trajectories of Alpha RBD_VCA (7LWS_VCA) during 200 ns MD simulation. **(A)** RMSD **(B)** RMSF **(C)** Radius of gyration of Protein **(D)** Radius of gyration of Complex **(E)** H-Bonds **(F)** SASA **(G)** Eigen values f the covariance matrix and **(H)** 2D Projection of trajectory.

**Fig. 10:**
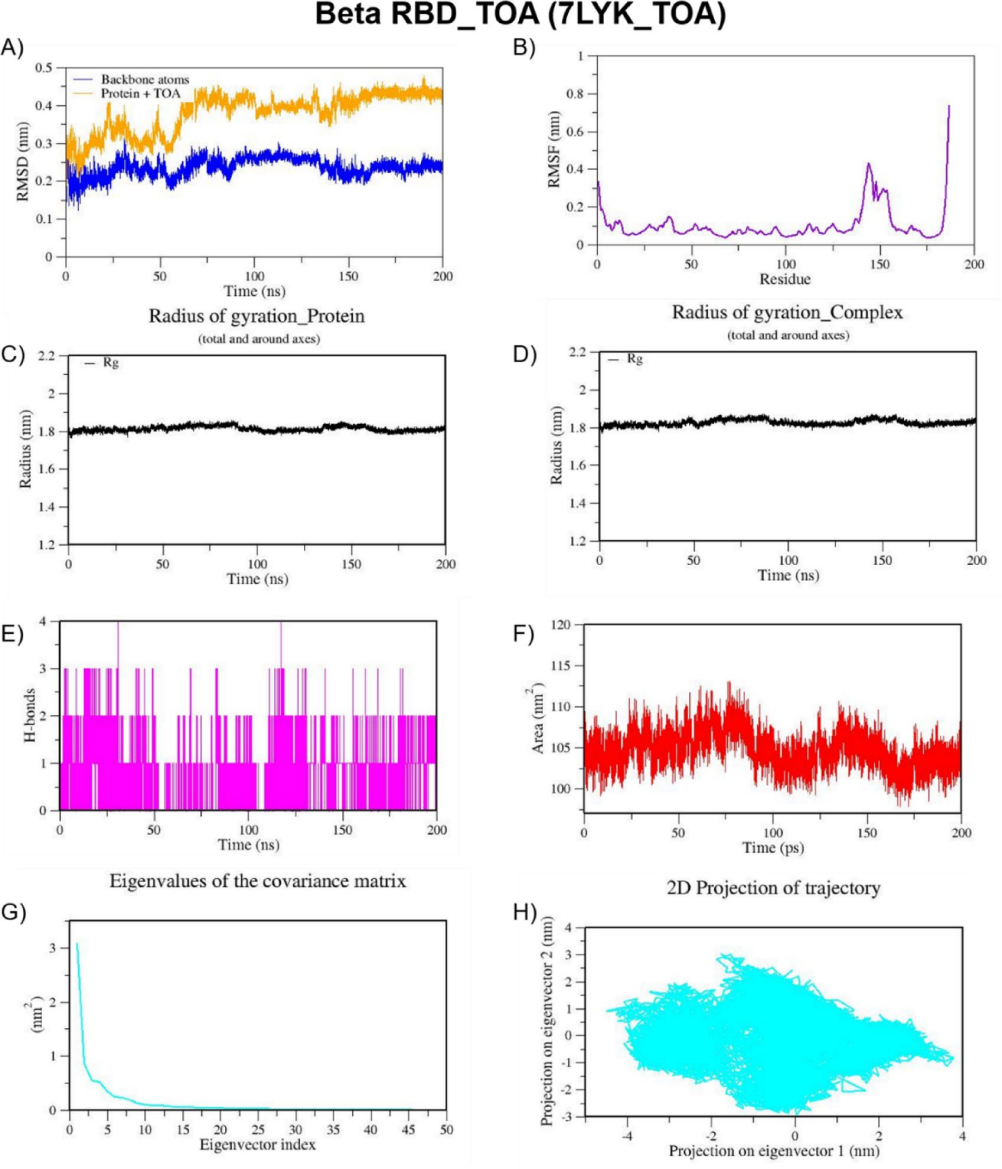
MD trajectories of Beta RBD_TOA (7LYK_TOA) during 200 ns MD simulation. **(A)** RMSD **(B)** RMSF **(C)** Radius of gyration of Protein **(D)** Radius of gyration of Complex **(E)** H-Bonds **(F)** SASA **(G)** Eigen values f the covariance matrix and **(H)** 2D Projection of trajectory.

**Fig. 11:**
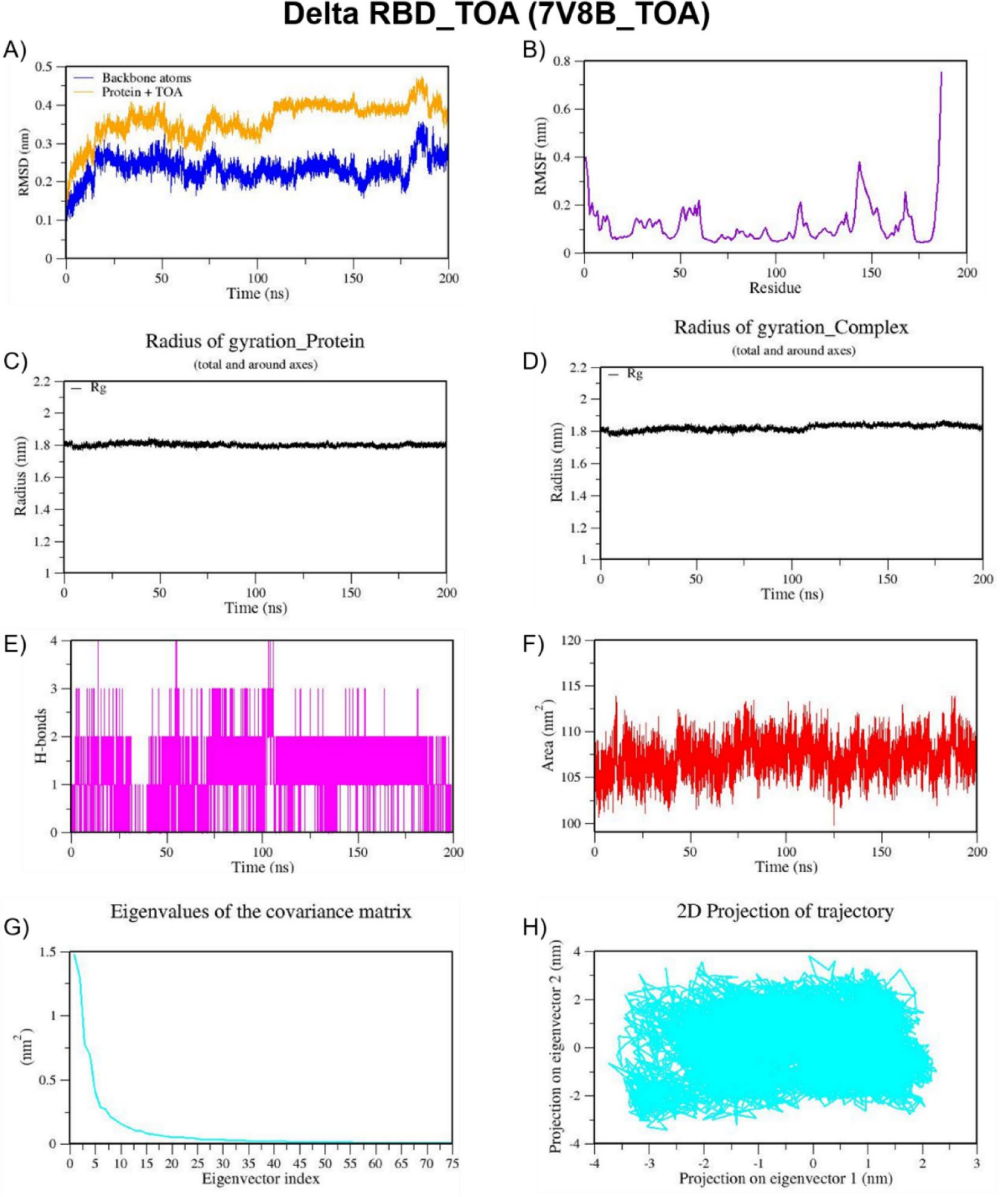
MD trajectories of Delta RBD_TOA (7V8B_TOA) during 200 ns MD simulation. **(A)** RMSD **(B)** RMSF **(C)** Radius of gyration of Protein **(D)** Radius of gyration of Complex **(E)** H-Bonds **(F)** SASA **(G)** Eigen values f the covariance matrix and **(H)** 2D Projection of trajectory.

**Fig. 12:**
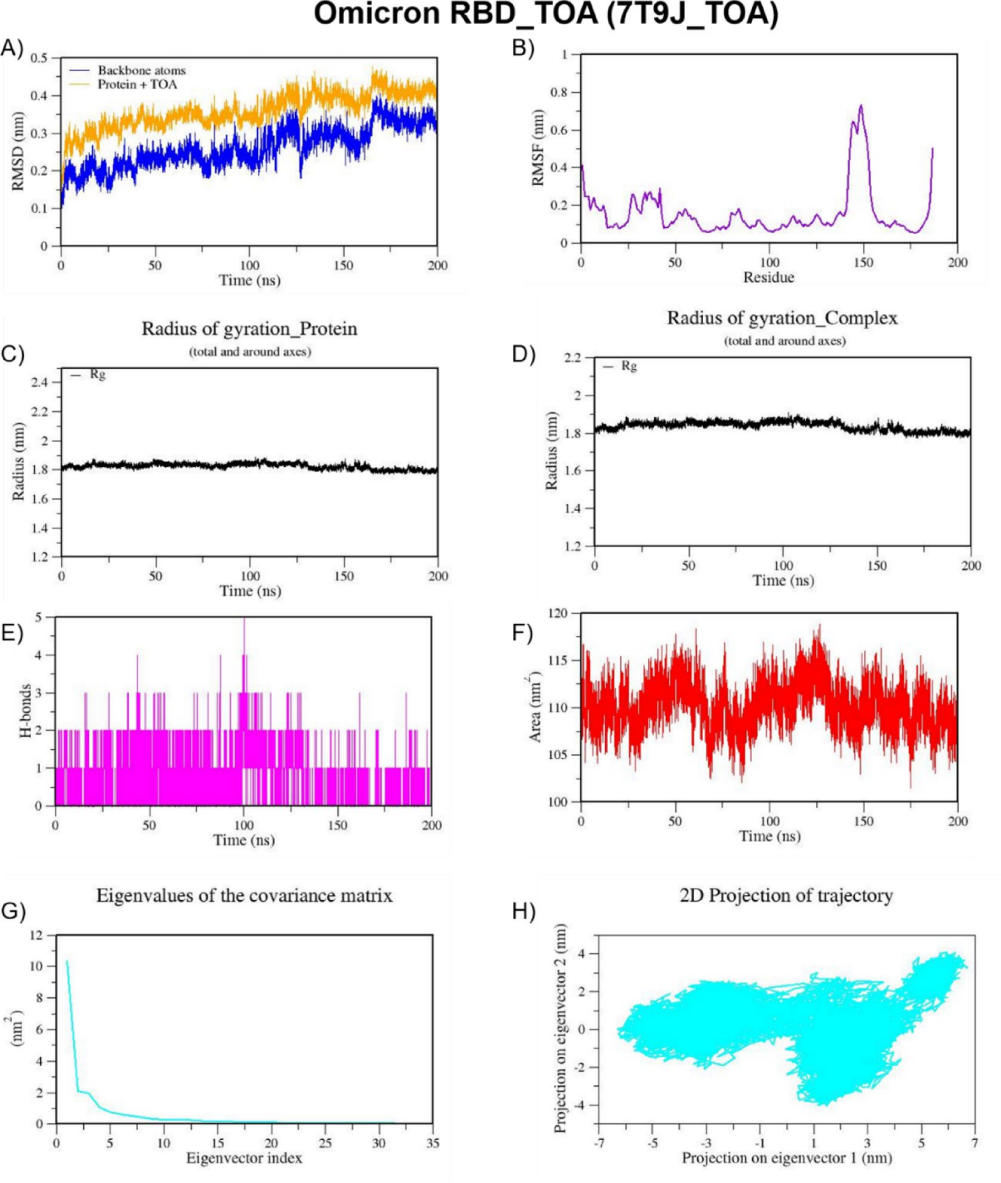
MD trajectories of Omicron RBD_TOA (7T9J_TOA) during 200 ns MD simulation. **(A)** RMSD **(B)** RMSF **(C)** Radius of gyration of Protein **(D)** Radius of gyration of Complex **(E)** H-Bonds **(F)** SASA **(G)** Eigen values f the covariance matrix and **(H)** 2D Projection of trajectory.

**Fig. 13:**
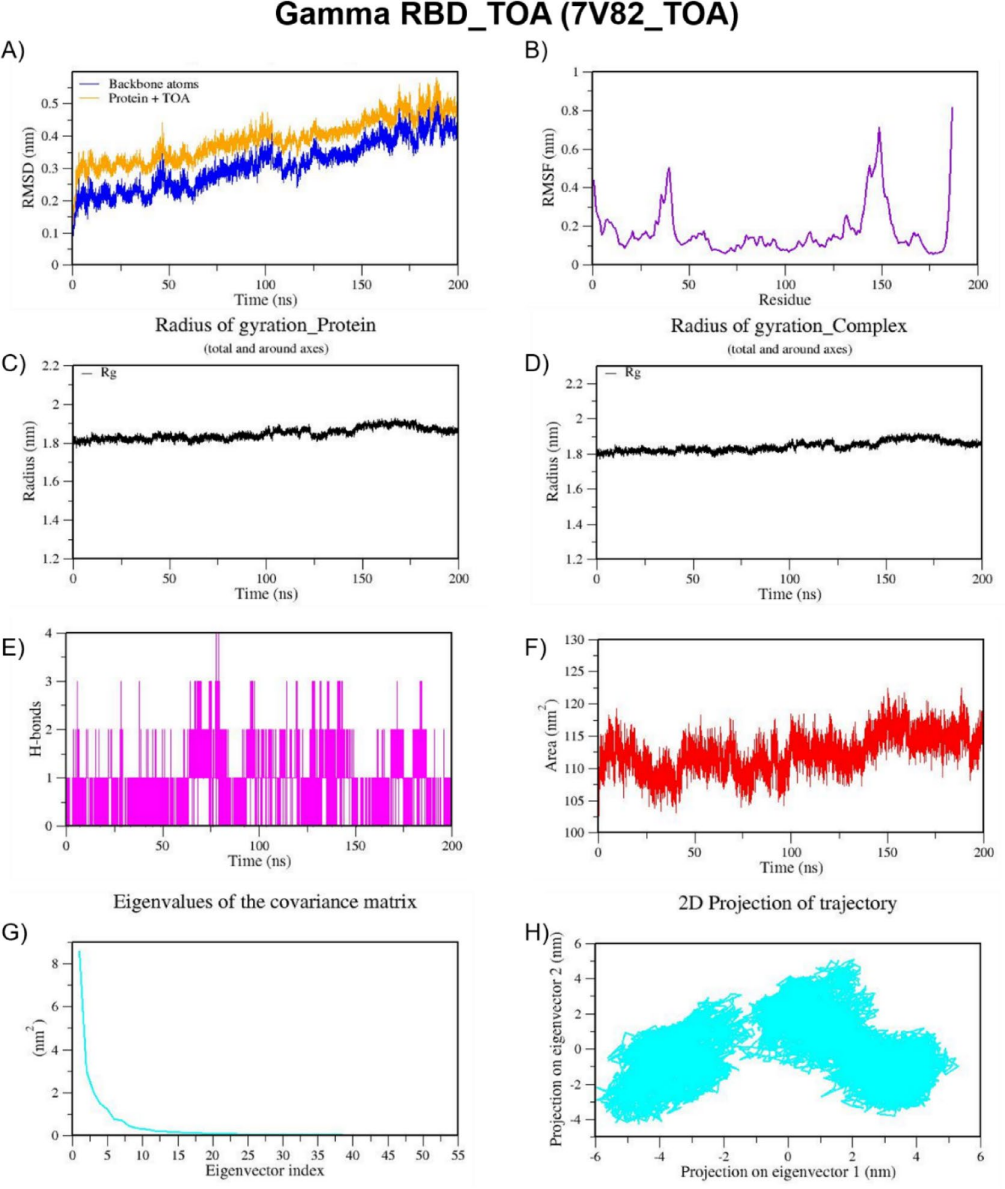
MD trajectories of Gamma RBD_TOA (7V82_TOA) during 200 ns MD simulation. **(A)** RMSD **(B)** RMSF **(C)** Radius of gyration of Protein **(D)** Radius of gyration of Complex **(E)** H-Bonds **(F)** SASA **(G)** Eigen values f the covariance matrix and **(H)** 2D Projection of trajectory.

We also performed MD simulation for the best docked complexes of reference compounds with the RBD of the spike from VOCs. For the MD simulation of the best docked compounds with VOCs, only the folded region observed between 334–520 amino acids of the S-protein from each VOC was selected. This region corresponds to the RBD domain and it does not consist of any missing residues. The top two docked compounds against the Alpha variant (PDB ID: 7LWS) include VCA (−8.4 Kcal/mol) and APO (−8.3 Kcal/mol). Here, we observed dominant hydrophobic interactions of VCA with the RBD domain of the S-protein of Alpha variant compared to other docked complexes. Interestingly, the physicochemical properties of VCA are excellent and falls within acceptable range when compared to APO. Therefore, MD simulation analysis has been carried out for the VCA-7LWS complex using the similar protocol used for previous MD simulation. Our docking analysis confirmed that TOA is the best docked compound against the RBD of S-protein from four VOCs such as the Beta, Delta, Omicron, and Gamma variants. In the case of the Beta variant, both TOA and VCA showed similar binding affinities and the same number of H-bonds (two). However, H-bonds shared by VCA exhibit shorter length than those of TOA. Hydrophobic interactions are crucial for stabilizing the ligand–protein complex, especially in the RBD of the S-protein, which has several hydrophobic patches. As result, TOA-7LYK complex has been selected for the MD simulation. The number of acceptable stereoisomers for a lead compound should be in the range of 0 to 5. However, this number is 0 for TOA, whereas APO has more than 5 stereocenters in its structure. This is the reason for including TOA-7LYK complex for MD simulation rather than the APO-7LYK. As evident from docking results, TOA showed the best mutation coverage in the RBD of Omicron (PDB ID: 7T9J). TOA interacts with several Omicron-specific mutated residues including ARG493, TYR453, TYR495, TYR501, and HIS505 in the RBD of S-protein. These mutations are critical for Omicron’s altered ACE2 binding capacity and immune escape. Therefore, TOA-7T9J complex has been selected for MD simulation. Whereas, TOA-7V8B complex was selected as TOA formed more hydrogen and hydrophobic contacts with key residues of RBD of the Delta variant. Likewise, TOA-7V82 complex has been chosen as TOA formed hydrogen and hydrophobic contacts with key residues of RBD of Gamma variant. MD trajectories of Alpha RBD_VCA (7LWS_VCA), Beta RBD_TOA (7LYK_TOA), Delta RBD_TOA (7V8B_TOA), Omicron RBD_TOA (7T9J_TOA), and Gamma RBD_TOA (7V82_TOA), were shown in Figs. 9, 10, 11, 12, and 13, respectively. From these figures, it is evident that MNPs from soft coral-derived microbes, particularly, VCA and TOA showed stable interactions with the RBD of the S-protein from VOCs.

## Discussion

The SARS-CoV-2 infections are still haunting and pose a challenge to develop effective antiviral drugs against this life-threatening viral infections^21^. Currently, available antiviral treatments are associated with side effects^26^. Therefore, it is necessary to develop cost-effective natural drugs with stable efficacy for COVID-19^27^. Computational approaches like molecular docking have always been invaluable tools for the swift screening of a large number of natural products and the discovery of lead molecules^22,23^. In this study, we for the first time, performed molecular docking of MNPs from soft coral-derived microbes against both S-protein and Mpro, the two key therapeutic target proteins of SARS-CoV-2. Our docking results revealed that MNPs such as Alterporriol O, Alterporriol Q, Fumiquinazoline K, Cottoquinazoline B/C/D, Tetraorcinol A, Versicoloritide A and C exhibited strong binding affinity towards the tested target proteins of SARS-CoV-2, with higher docking scores compared to positive control ligands (nelfinavir and remdesivir) (Fig. 2). Moreover, these compounds have been shown to exhibit strong binding affinities towards the RBD of five SARS-CoV-2 mutants. Although previous studies showed that remdesivir and isoquercetin can inhibit the MTase activity of several flaviviruses^36^, the main concern about their therapeutic use is the toxicity associated with the particular drug. From our docking results, it can be suggested that the best-docked compounds may inhibit spike protein and Mpro, thereby preventing the virus from spreading.

Diverse SARS-CoV-2 mutants have evolved due to point-mutations in the RBD of the S-protein. The most widely reported SARS-CoV-2 VOCs include Alpha (B1.1.7), Beta (B.1.351), Delta (B.1.617.2), Gamma (P.1), and Omicron (B.1.1.529) variants^20^. The Alpha variant constitutes the first VOC detected in the United Kingdom and it accounts for over 95% of COVID cases with 55% deaths in the UK. It consists of several mutations including deletions (H69, V70, and Y144) and one substitution of asparagine (N) with tyrosine (Y) (N501Y) in the S-protein. It has been reported that these mutations in the Alpha variant spike play a critical role in the enhanced kinetics of viral infection and replication compared to the WT virus^37^. Particularly, the N501Y mutation significantly contributes to the proliferation of viral particles and increased infectivity. This point substitution of N by Y, leads to the formation of an extra H-bond with LYS353 of hACE2 (LYS353-TYR501). This increases the electrostatic binding affinity of surface protein hACE2 to spike protein, therefore, strong binding of SARS-CoV-2 to the host surface^38^. Tian et al.’s MD simulation studies suggest that the Alpha variant shows stronger interaction than the WT type with the hACE2 receptor which is because this point mutation induces a decrease in the virus-cell dissociation constant^39^. Previous studies reported that the N501Y mutation promotes the S-protein’s open trimer prefusion conformation state, which leads to the exposure of the RBD that is buried in the prefusion conformation state of the trimeric S-protein. This open conformation state is necessary for the RBD to recognize and engage the hACE2 receptor^40,41^. The same effect has been predicted for other mutations on glycine residues (404, 416, 504) as well as at the K417 residue^41^. Only three among the top docked compounds, Alterporriol O, Versicoloritide A, and Versicoloritide C, interacted with TYR501, a key residue of the Alpha spike variant. Docking results showed that Alterporriol O formed several H-bonds and hydrophobic interactions via key amino acids GLN493, TYR501, and TYR505. Although Alterporrirol Q and Fumiquinazoline K formed few H-bonds and several hydrophobic contacts, they did not interact with TYR501. However, Versicoloritide A and Versicoloritide C formed H-bonds and hydrophobic interactions with TYR501 as well as with TYR505.

The Beta variant was first identified in South Africa and it accounted for about 28.8% of deaths. The Beta variant has been characterized by several important point mutations (N501Y, E484K, and K417N) in the RBD of the S-protein. The E484K mutation confers the Beta variant resistance against monoclonal antibodies that are targeted to its RBD^42,43^. The E484K in conjunction with K417N in the Beta variant, has been shown to reduce the ability of monoclonal antibodies to bind to the Beta variant’s RBD of the S-protein^44^. Other studies also reported that E484K in conjunction with D614G mutation induces the open prefusion conformation of spike trimer of the Beta variant, subsequently leading to an increase in the binding affinity to hACE2^45^. This more open conformation in the Beta variant S-protein prevents the salt bridge/H-bond network from forming, hence preventing the formation of the more closed conformation seen in the WT S-protein. Despite these conformational differences between the Beta variant and the WT in its binding characteristics to ACE2, the Beta variant does have lower fitness compared to the Alpha variant in terms of replication kinetics in human airway epithelial cells in culture. Furthermore, it has also been shown that the replicative capacity of the Beta variant was significantly lower than that of the Alpha or the progenitor variant when tested in hamster models^46^. In a K18-hACE2 transgenic mouse study, it was shown that Alpha and Beta variants were 100-fold more lethal than the WT^47^. It has been reported that the Beta (B.1.351) variant, particularly the variant with the N501Y mutation, is associated with increased transmissibility^39^.

The Delta variant was one of the most prominent VOCs to have emerged in India. It is 40%–60% more transmissible than the Alpha (B.1.1.7) variant and may be associated with an increased risk of hospitalization. In addition to the P681R mutation in the furin cleavage site, this variant is also characterized by point mutations in S-protein’s RBD including T478K and L452R. These mutations are reduce protein stability and increase disease risk^48^. Our results showed that none of the best docked compounds could interact with T478K and L452R. However, they showed binding interactions with other amino acid residues such as GLY496, ASN501, TYR505, GLN498, LEU455, TYR453, LYS417, and PHE456. Tetraorcinol A was found to be the only best docked compound forming both H-bond and hydrophobic interactions with most of the amino acids of the RBD of the Delta variant spike protein.

The Omicron variant harbors about 15 mutations in the RBD of spike. This variant also shares several mutations with the Alpha, Beta, and Gamma variants^49^. The point-mutations such as N501Y, K417N, Q493R, and T478K were among several others that have been reported. Despite high binding affinities of Cottoquinazoline Q and D towards the Omicron spike variant’s RBD, they did not significantly interact with any of the key residues of the RBD of Omicron variant. Only Tetraorcinol A was found to form significant hydrophobic interactions with the RBD’s key residues such as ARG493, TYR501 and HIS505. Alterporriol Q formed one H-bond and one hydrophobic interaction with ARG493 but not with any other key mutated residues.

Gamma variant was initially discovered in Brazil and it contains 17 mutations including three-point mutations (K417T, E484K, and N501Y) that were observed in the RBD of the S-protein^50^. These mutations have been associated with enhanced binding to hACE2. The top 5 docked compounds against the Gamma spike variant’s RBD include Alterporrirol Q, Tetraorcinol A, Versicoloritide A, Alterporrirol P, and Fumiquinazoline K. We noticed two compounds, Alterporrirol Q, despite its high binding energy, and Alterporriol P, did not interact with any of the three key mutant residues of the RBD of the Gamma variant. However, Tetraorcinol A formed H-bonds with key amino acids GLN493 and LYS484 and formed several hydrophobic interactions with other RBD’ key residues TYR489, PHE456, TYR489, LYS484, LYS484, and LYS484. Versicoloritide A and Fumiquinazoline K formed one hydrogen bond with GLN493 and TYR501, respectively. Versicoloritide A also formed hydrophobic contacts, two with TYR505 and one with TYR501. Similarly, Fumiquinazoline K formed hydrophobic contacts, two with TYR505 and two with TYR501.

The compounds of this study exhibit diverse pharmacokinetic and pharmacodynamic properties. Notably, cyclic pentapeptides such as Versicoloritides A, B, and C demonstrate strong P-glycoprotein (Pgp) inhibition, which is consistent with previous findings that certain fungal peptides can modulate drug efflux pumps^51,52^, potentially overcoming multidrug resistance infectious diseases. However, their high CYP3A4 inhibitory activity raises concerns about drug-drug interactions, a phenomenon well-documented with other natural Pgp inhibitors like cyclosporine and ketoconazole^53^. Additionally, the violation of Lipinski’s rule by Versicoloritide C suggests limited oral bioavailability, a common challenge with peptide-like fungal metabolites that may require structural optimization or alternative delivery systems^54^.

The Cottoquinazolines (B, C, and D) emerge as promising drug-like candidates due to their favorable Lipinski compliance, moderate Pgp inhibition, and low mutagenic risk (Ames-test). These findings correlate with prior studies on quinazoline alkaloids, which have shown antimicrobial and anticancer activities with relatively low toxicity^55^. The moderate Pgp inhibition of Cottoquinazoline-C (0.723) suggests a potential role in combination therapies to enhance chemotherapeutic efficacy, similar to the synthetic Pgp inhibitor tariquidar^56^. However, their relatively low Caco-2 permeability (−5.371 to −5.553) indicates possible absorption limitations, a hurdle that has been addressed in related compounds through prodrug strategies or nanoformulations^57^.

Tetraorcinol A (TOA) is an orcinol tetramer that belongs to the class of polyketides^58^. Orcinol and its derivatives have been shown to exhibit biological effects including antioxidant, anti-inflammatory, and anti-cancer activities. Diorcinol is the simplest diphenyl ether secreted as secondary metabolite of a variety of fungi and it has been shown to demonstrate significant antimicrobial and antitumor activity^59^. Tetraorcinol A shares structural similarity to orcinol and Diorcinol. ADMET analysis predicts that TOA obeys Lipinski rule of five and it has a PAINS value of 0. This implies that TOA has no known interference motifs. From our molecular docking and MD simulation results, it can be suggested the possible antiviral potential of TOA, owing to the presence of several orcinol units in its structure.

Aspergillipeptide C and the Alterporriol (Q and P) exhibit serious disadvantages, such as PAINS alerts and poor drug-likeness, which are in line with research reports on the risk of assay interference and promiscuity associated with some polyketides and peptides^60^. Although Alterporriol O exhibits notable Pgp inhibition (0.855), its PAINS flag suggests potential off-target effects, limiting its therapeutic potential. Reiterating earlier findings that certain fungal-derived quinazolines may have genotoxic side effects, Fumiquinazoline-K also shows good oral bioavailability predictions (ROA: 0.919) but carries carcinogenicity concerns^61^. These results highlight how crucial it is to maintain a balance between bioactivity and safety when developing new natural product-based drugs, requiring additional structural improvement or mechanistic research to reduce risks while maintaining therapeutic benefits.

Phytosterols have also been demonstrated to possess antiviral activity against the spike protein of the COVID-19 virus and influenza-A virus^31^. Our study identified compounds such as Alterporriol Q, Cottoquinazoline C and Versicoloritide A, demonstrating potent inhibitory affinity. Previous in silico studies have also demonstrated that anthraquinones such as Alterporriol Q could act as a potential inhibitor of Mpro of SARS-CoV-2^62^. The most essential step in drug development is to perform in silico analyses on compounds to assess their ADMET properties. Failure to accurately simulate these attributes or assess any toxicities may cause inhibitors to fail the screening process and thus fall outside the criteria for approval. An important consideration when developing drugs to treat central nervous system (CNS) conditions is BBB penetration^63^. Cytochrome P450 (CYP) isozymes metabolize drugs, fatty acids, steroids, bile acids, and carcinogens^64^ Approximately 75% of phase-1 drug metabolism processes involve CYP enzymes^65^. Various CYP inhibitor and substrate scores were calculated in this analysis, and the results show that the shortlisted compounds are non-inhibitors of CYP enzymes (Table 4). Our ADME analysis demonstrated that most of the best docked compounds had non-toxic properties while Lipinski’s rule of five was accepted by 8 of the top docked compounds.

## Conclusion

Our in-silico virtual screening results from molecular docking and MD simulation reveal that several MNPs from soft coral-derived microbes showed strong binding affinity towards SARS-CoV-2 Mpro and the RBD of S-protein from both WT and VOCs. Our ADMET analysis together with MD simulations collectively suggest the possible antiviral potential test compounds against the SARS-CoV-2. This implies that MNPs (Cottoquinazoline B, Tetraorcinol A, and Versicoloritide A) from soft coral-derived microbes may have the potential to develop as antiviral medicines for COVID-19. However, further experimental validations are still required to potentially evaluate their antiviral activity.

## Supplementary Information

Below is the link to the electronic supplementary material.

Supplementary Material 1

Supplementary Material 2

## Data Availability

The datasets used and/or analysed during the current study available from the corresponding author on reasonable request.
