# Supplementary material for: Molecular docking and dynamic simulation of marine natural products from soft coral-derived microbes against SARS-CoV-2 main protease and spike protein
Source: Sci Rep. 2026 Feb 11;16:8252. doi: 10.1038/s41598-026-37446-6 (PMC12963565; doi:10.1038/s41598-026-37446-6)
Supplement: Supplementary file 1 — Supplementary Material 1 [file 41598_2026_37446_MOESM1_ESM.docx]

**Supplementary Fig 1.** Binding mode of the top five best docked coral reef compounds against RBD of spike from SARS Co V-2 Variants of Concern (VOCs) (shown in 3D) and with the prediction of 2D stabilized intermolecular interactions of docking studies.

| 7LWS-APO Complex (Alpha RBD-APO complex) | | |  |
| --- | --- | --- | --- |
| 3D | | 2D |  |
| 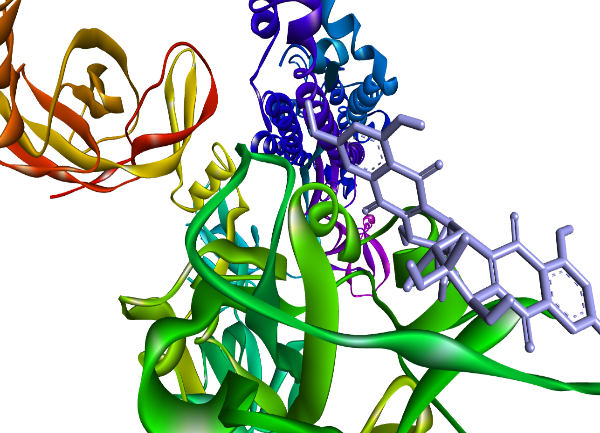 | | 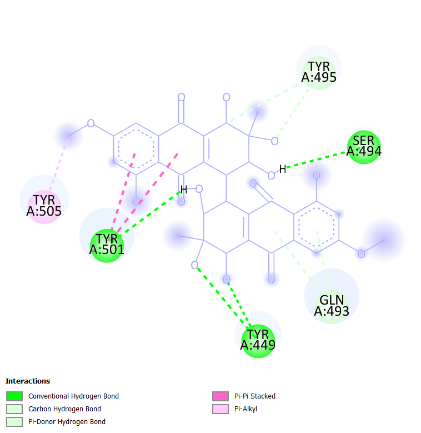 |  |
| 7LWS-APQ Complex (Alpha RBD-APQ complex) | | |  |
| 3D | | 2D |  |
| 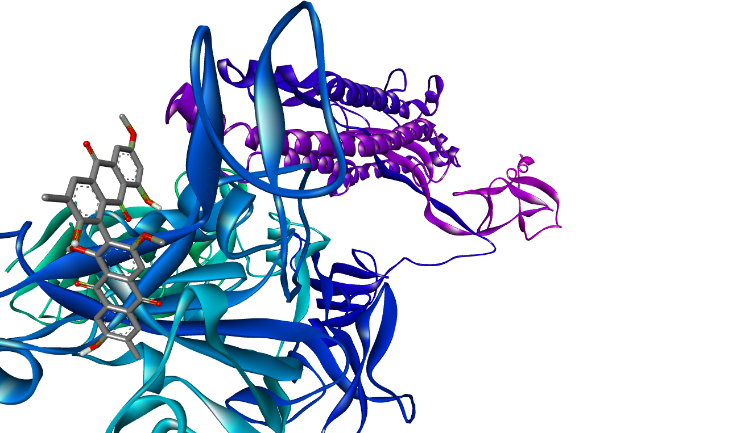 | | 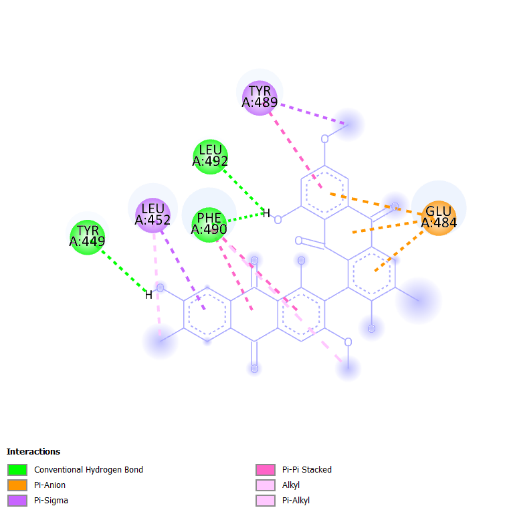 |  |
| 7LWS-FUKComplex (Alpha RBD-FUK complex) | | |  |
| 3D | | 2D |  |
| 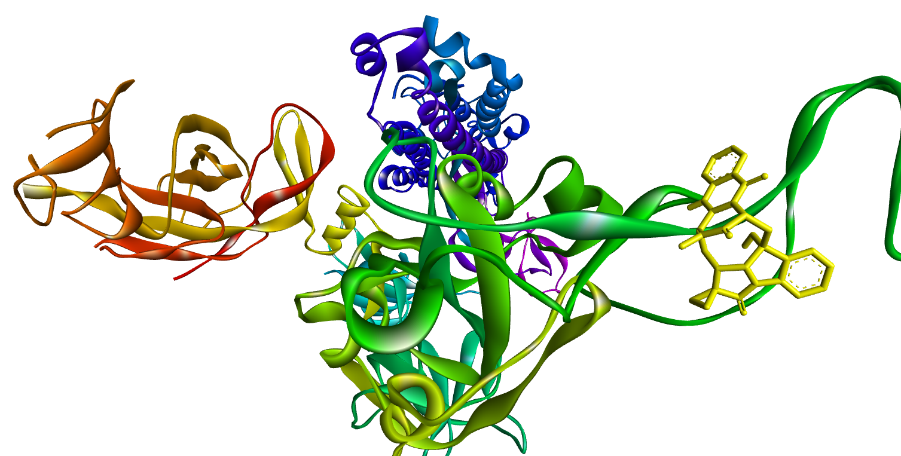 | | 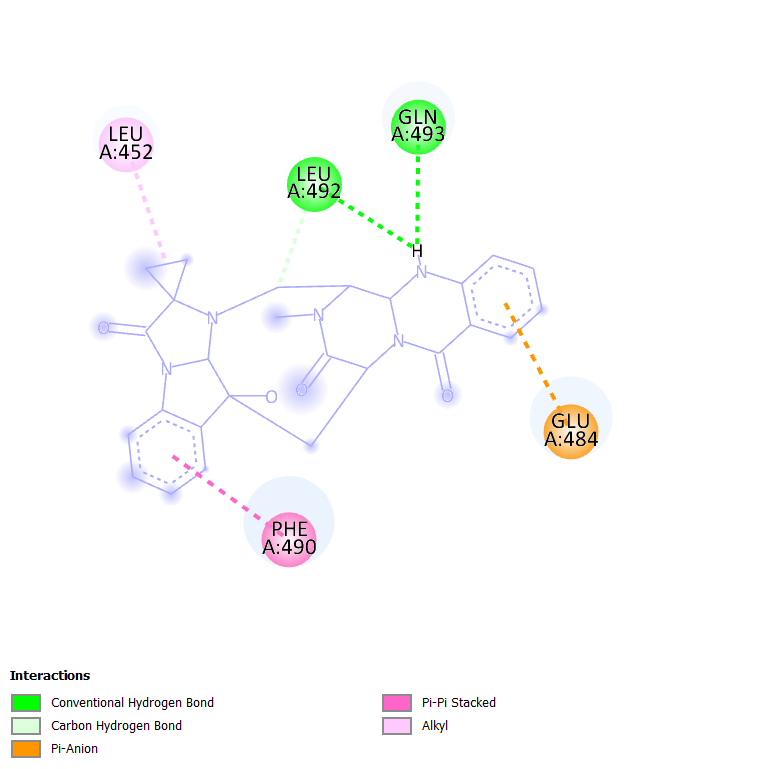 |  |
| 7LWS-VCAComplex (Alpha RBD-VCA complex) | | |  |
| 3D | 2D | |  |
| 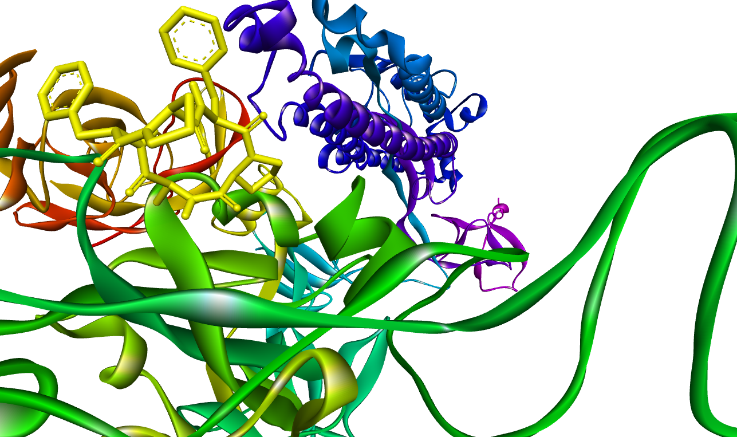 | 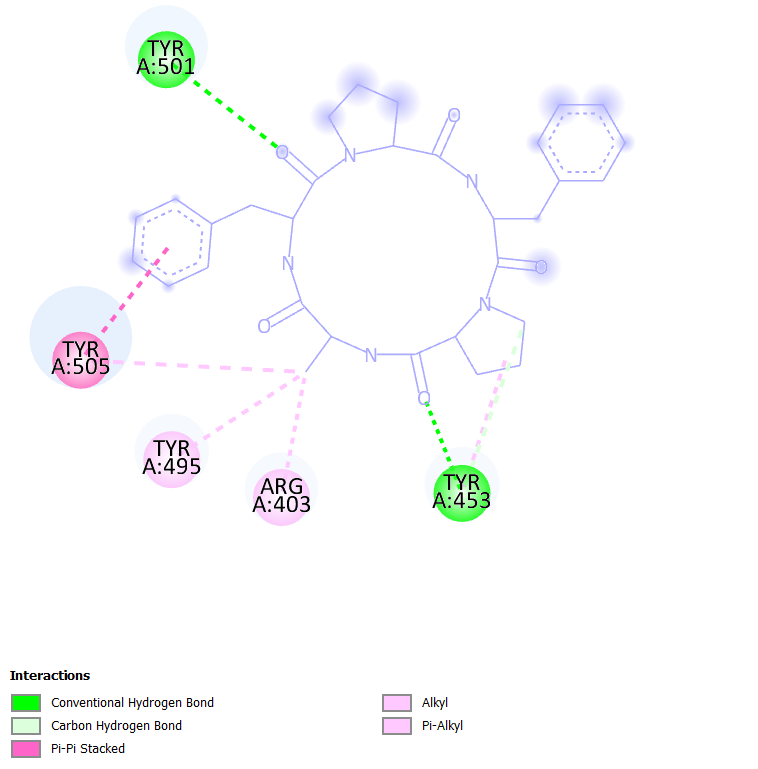 | |  |
| 7LWS-VCC Complex (Alpha RBD-VCC complex) | | |  |
| 3D | 2D | |  |
| 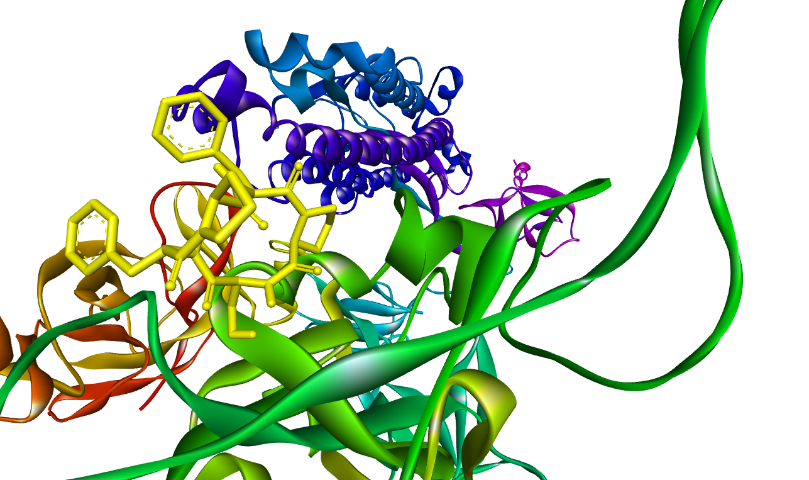 | 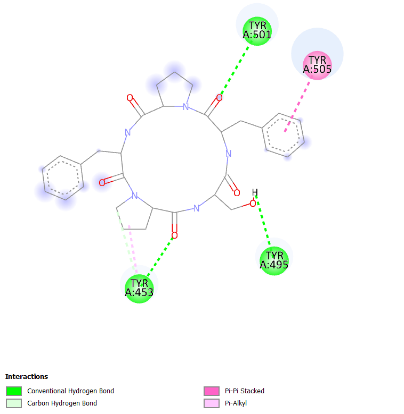 | |  |
| 7LYK-CQB Complex (Beta RBD-CQB Complex) | | |  |
| 3D | 2D | |  |
| 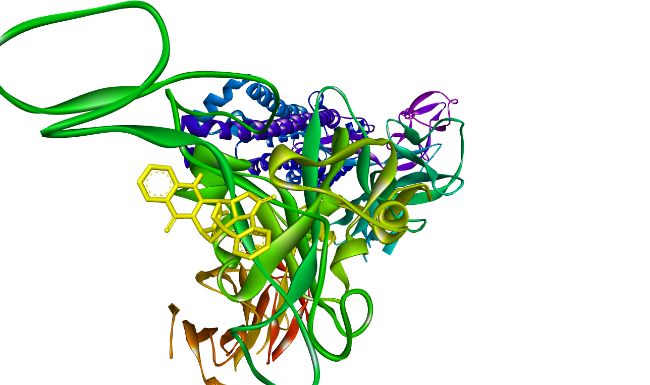 | 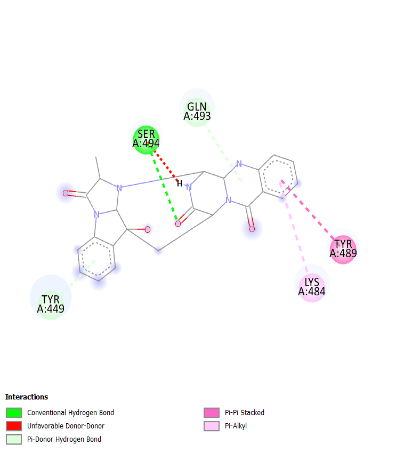 | |  |
| 7LYK-CQD Complex (Beta RBD-CQD Complex) | | | |
| 3D | | 2D | |
| 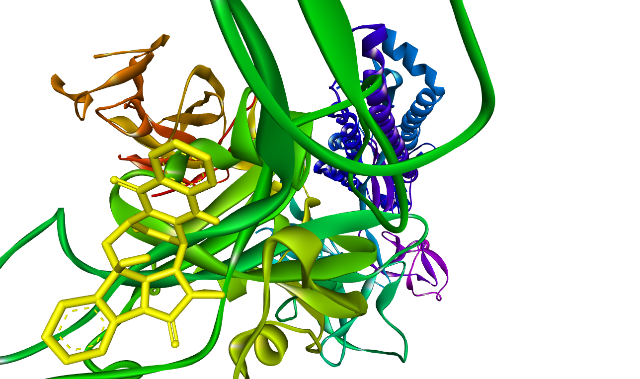 | | 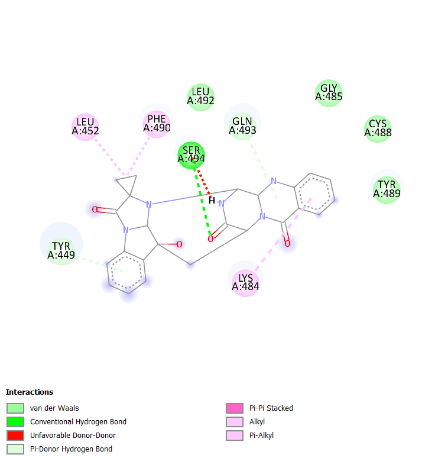 | |
| 7LYK-FUK Complex (Beta RBD-FUK Complex) | | | |
| 3D | | 2D | |
| 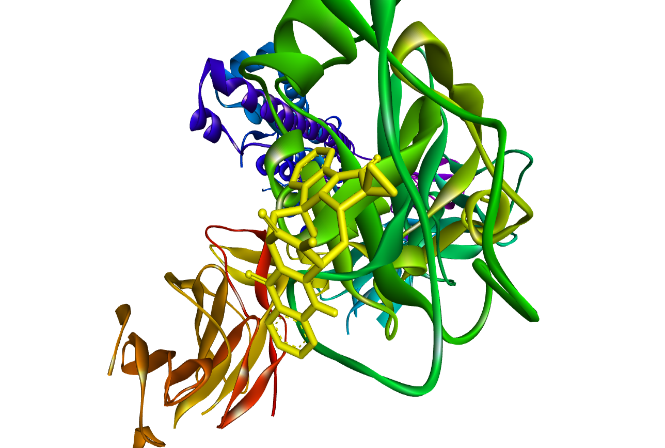 | | 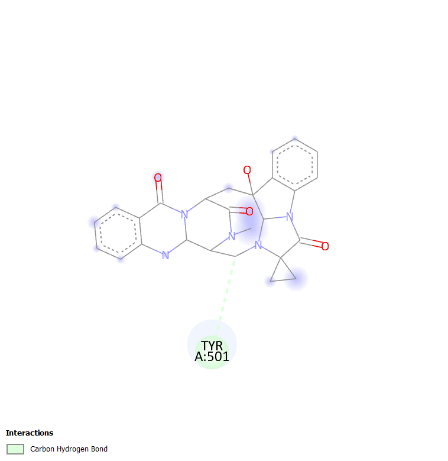 | |
| 7LYK-TOA Complex (Beta RBD-TOA Complex) | | | |
| 3D | | 2D | |
| 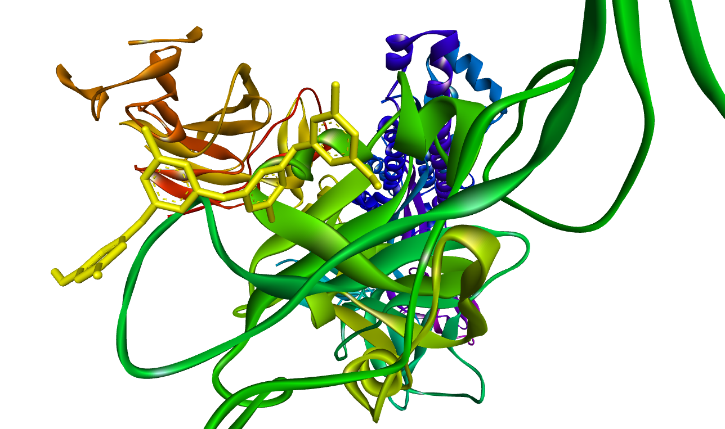 | | 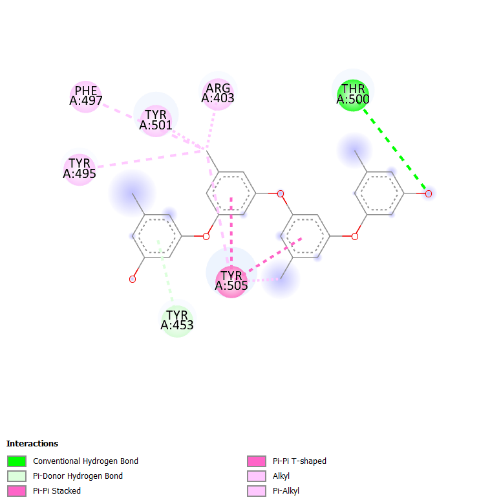 | |
| 7LYK-VCA Complex (Beta RBD-VCA Complex) | | | |
| 3D | | 2D | |
| 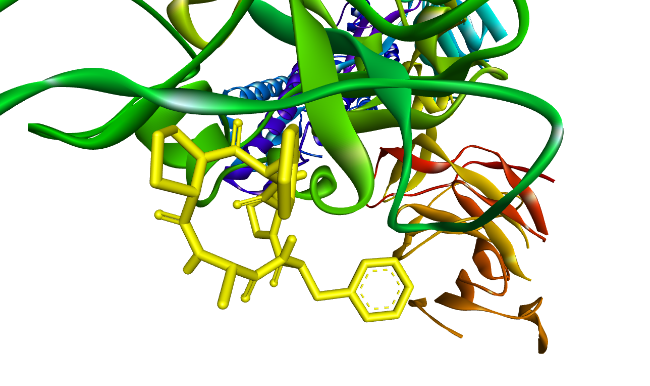 | | 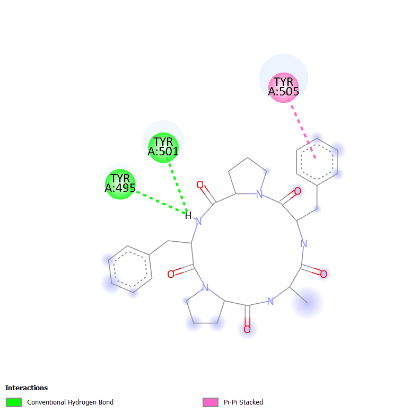 | |
| 7T9J-APQ Complex (Omicron RBD-APQ Complex) | | | |
| 3D | | 2D | |
| 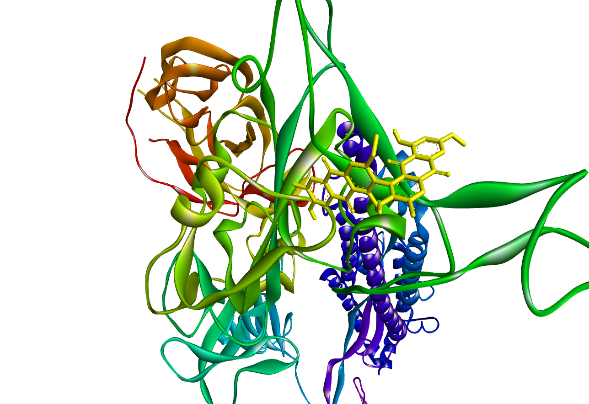 | | 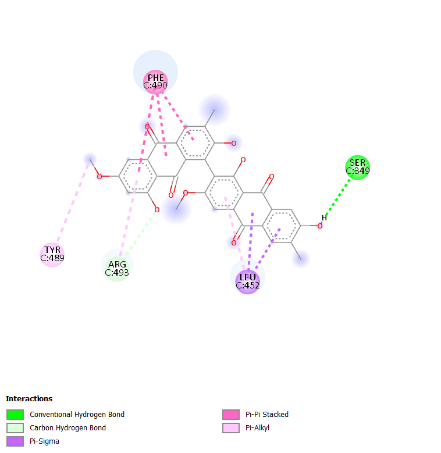 | |
| 7T9J-CQBComplex (Omicron RBD-CQB Complex) | | | |
| 3D | | 2D | |
| 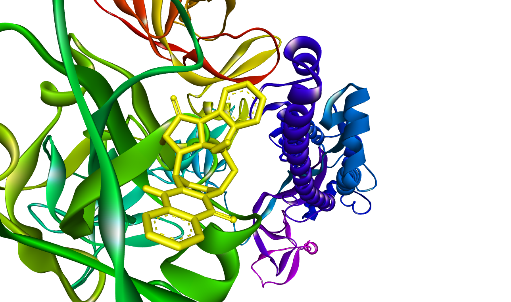 | | 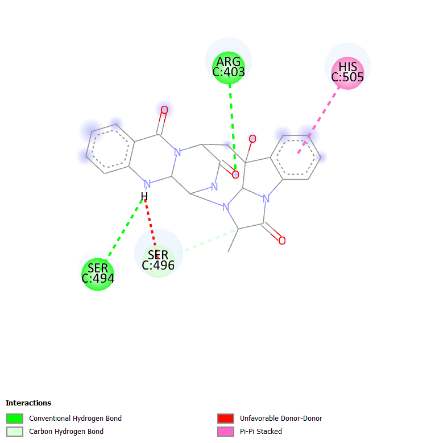 | |
| 7T9J-CQD Complex (Omicron RBD-CQD Complex) | | |  |
| 3D | 2D | |  |
| 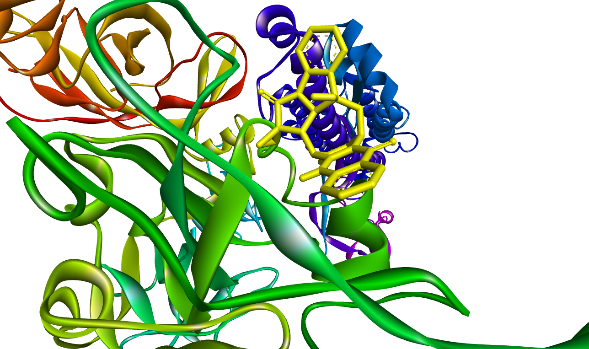 | 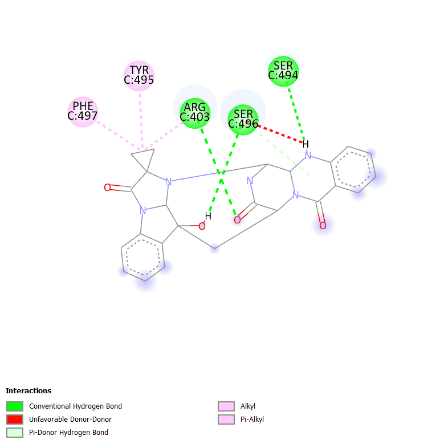 | |  |
| 7T9J- FUKComplex (Omicron RBD-FUK Complex) | | |  |
| 3D | 2D | |  |
| 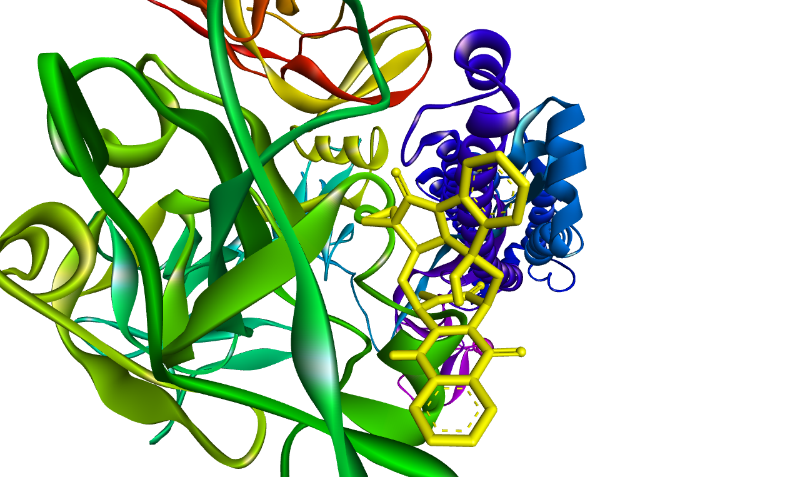 | 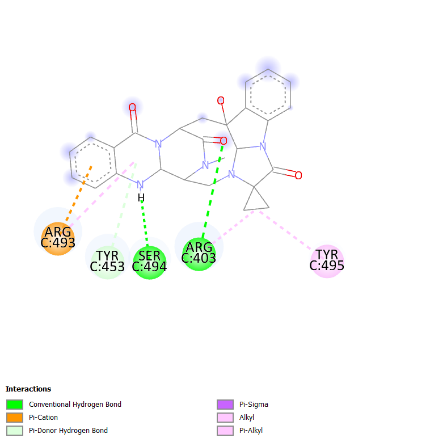 | |  |
| 7T9J- TOA Complex ((Omicron RBD-TOA Complex) | | |  |
| 3D | 2D | |  |
| 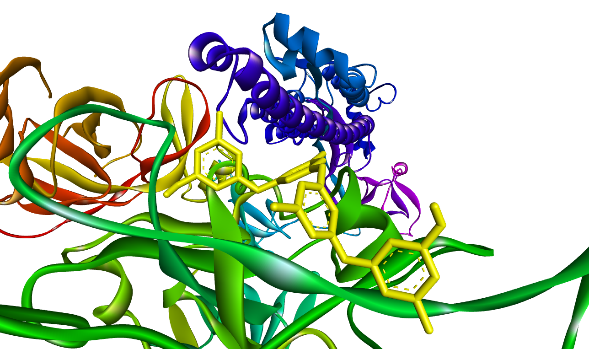 | 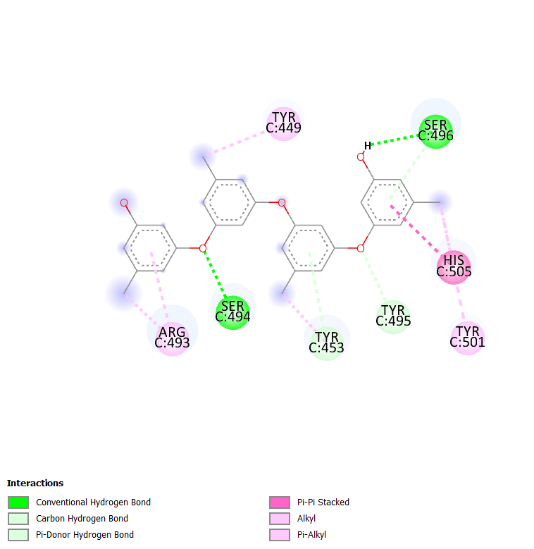 | |  |
| 7V8B – CQB Complex (Delta RBD- CQB Complex) | | |  |
| 3D | 2D | |  |
| 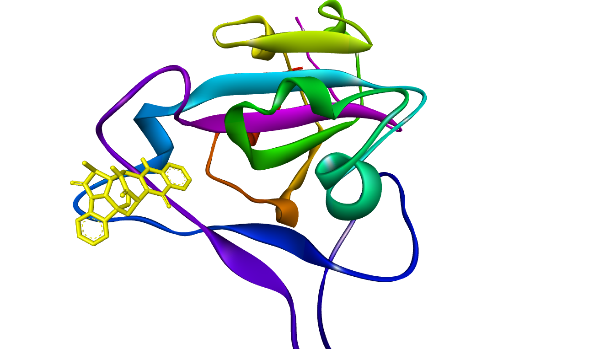 | 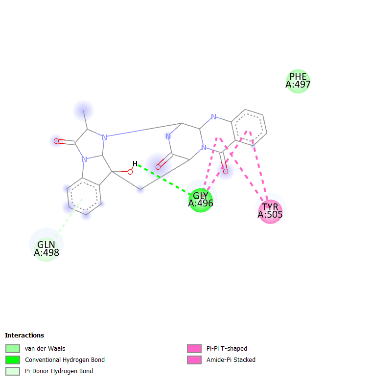 | |  |
| 7V8B- CQD Complex (Delta RBD- CQD Complex) | | |  |
| 3D | 2D | |  |
| 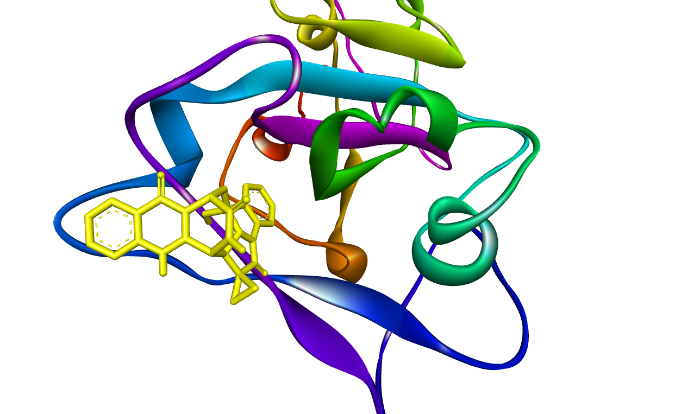 | 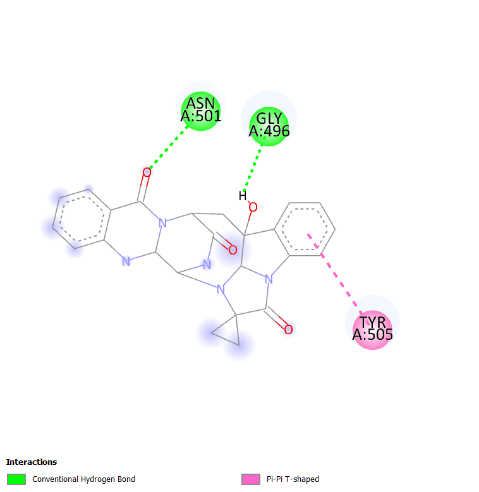 | |  |
| 7V8B - FUK Complex (Delta RBD- FUK Complex) | | |  |
| 3D | 2D | |  |
| 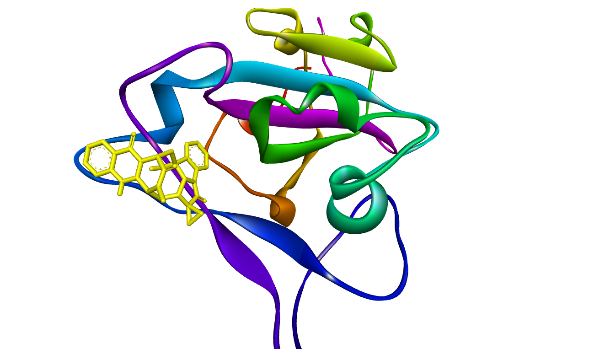 | 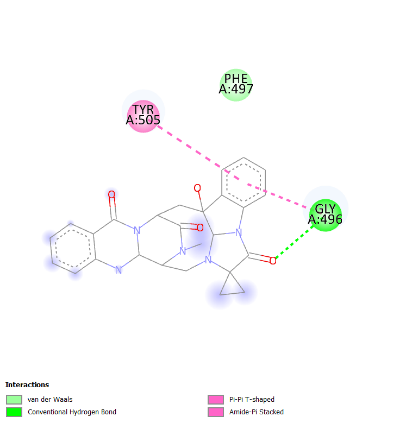 | |  |
| 7V8B - TOA Complex (Delta RBD- TOA Complex) | | |  |
| 3D | 2D | |  |
| 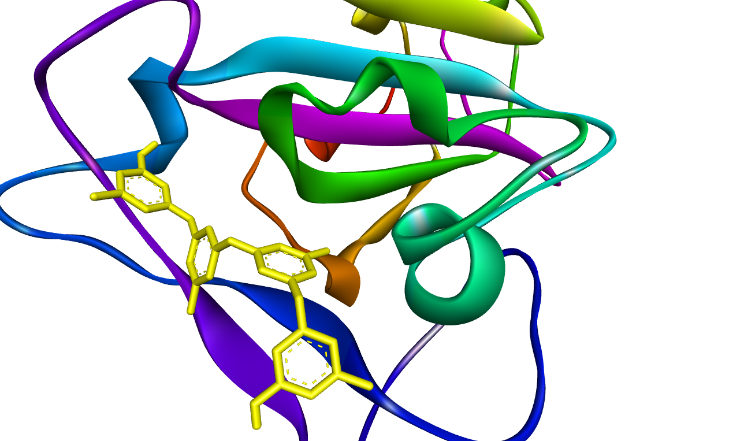 | 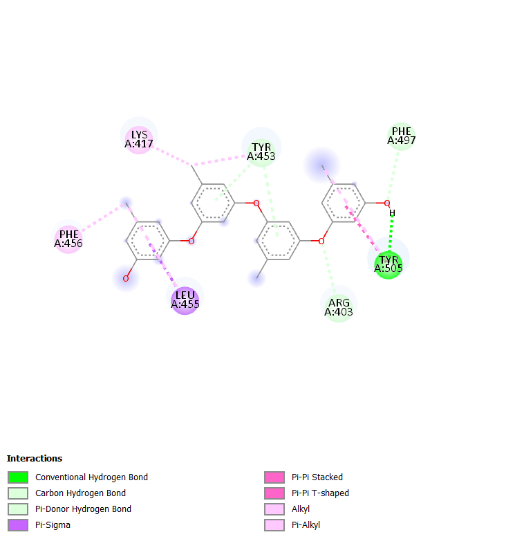 | |  |
| 7V8B-VCB Complex (Delta RBD- VCB Complex) | | |  |
| 3D | 2D | |  |
| 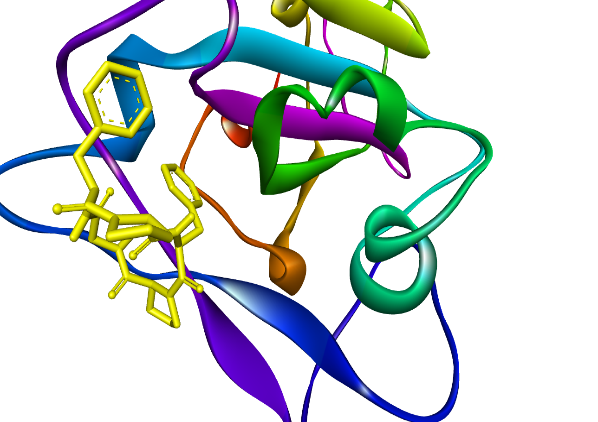 | 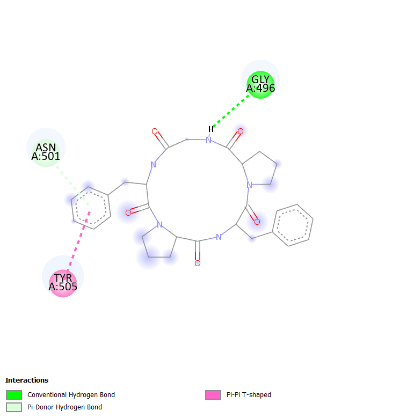 | |  |
| 7V82-APP Complex (Gamma RBD-APP Complex) | | |  |
| 3D | 3D | |  |
| 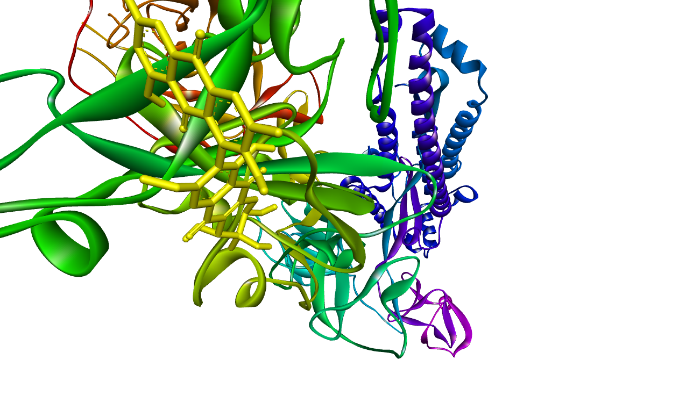 | 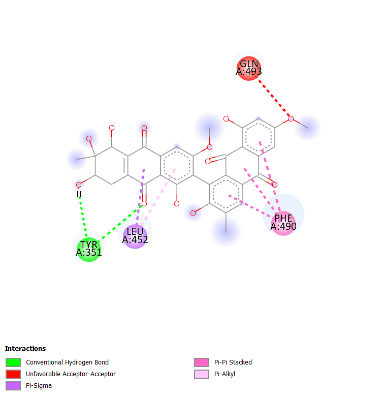 | |  |
| 7V82- APQ Complex (Gamma RBD-APQ Complex) | | |  |
| 3D | 3D | |  |
| 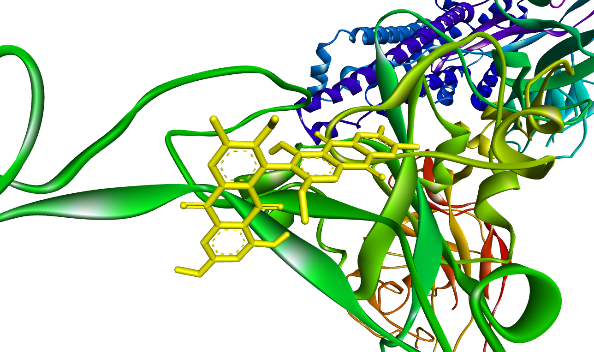 | 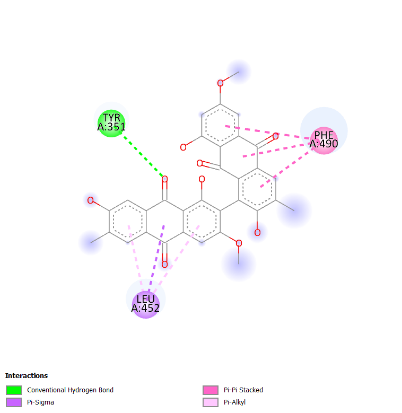 | |  |
| 7V82- TOA Complex (Gamma RBD-TOA Complex) | | |  |
| 3D | 3D | |  |
| 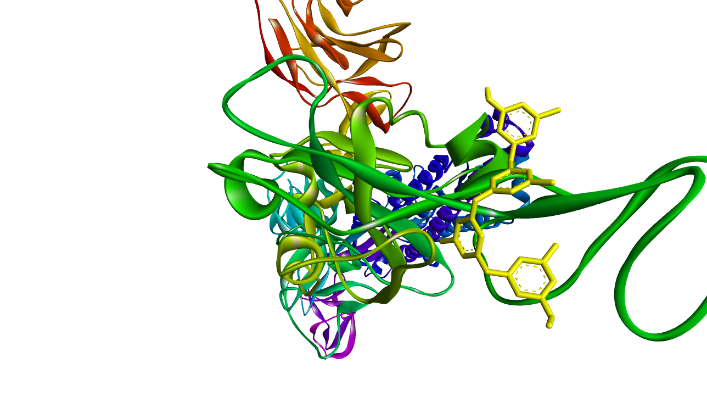 | 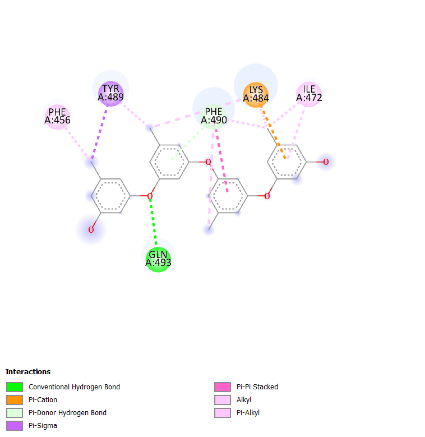 | |  |
| 7V82 -FUK Complex (Gamma RBD-FUK Complex) | | |  |
| 3D | 3D | |  |
| 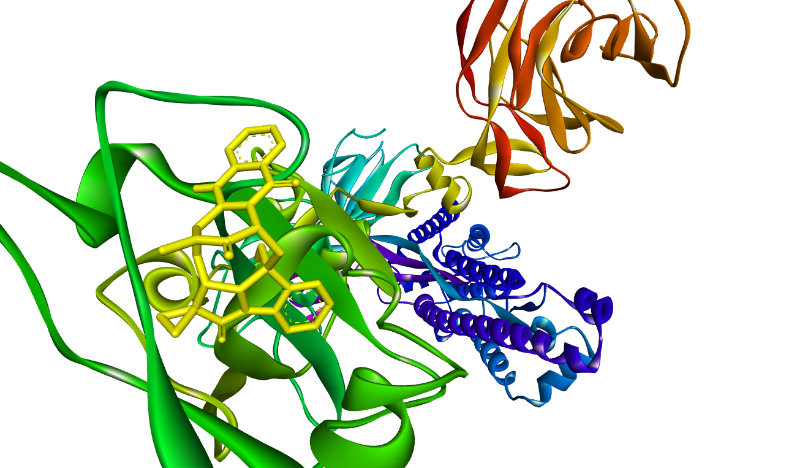 | 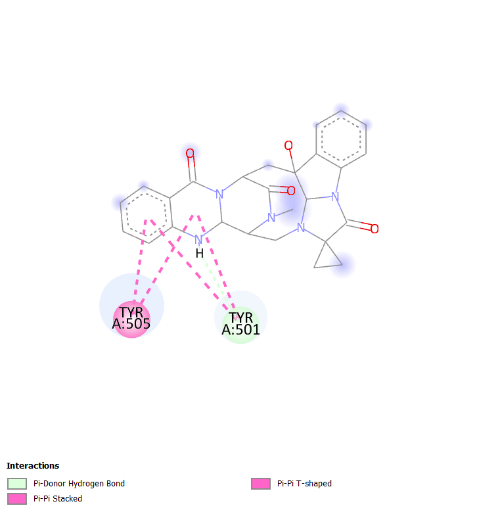 | |  |
| 7V82 - VCA Complex (Gamma RBD-VCA Complex) | | |  |
| 3D | 3D | |  |
| 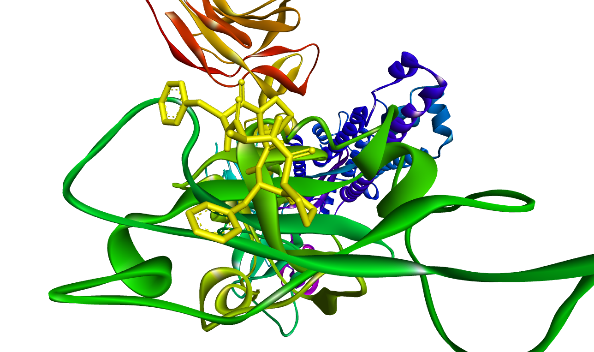 | 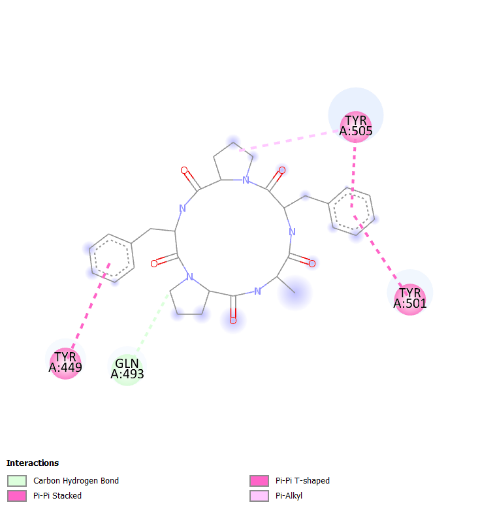 | |  |
